# Disruptions in outer membrane-peptidoglycan interactions enhance bile salt resistance in O-antigen-producing *E. coli*

**DOI:** 10.1128/mbio.02184-25

**Published:** 2025-08-28

**Authors:** Jilong Qin, Yaoqin Hong, Waldemar Vollmer, Renato Morona, Makrina Totsika

**Affiliations:** 1 Centre for Immunology and Infection Control, School of Biomedical Sciences, Queensland University of Technology 1969, Brisbane, Queensland, Australia; 2 Max Planck Queensland Centre, Queensland University of Technology 1969, Brisbane, Queensland, Australia; 3 Institute for Molecular Bioscience, The University of Queensland 1974, Brisbane, Queensland, Australia; 4 Biomedical Sciences and Molecular Biology, College of Medicine and Dentistry, James Cook University 8001, Townsville City, Queensland, Australia; 5 Centre for Bacterial Cell Biology, Biosciences Institute, Newcastle University 5994, Newcastle upon Tyne, United Kingdom; 6 School of Biological Sciences, Department of Molecular & Biomedical Sciences, Research Centre for Infectious Diseases, University of Adelaide 1066, Adelaide, South Australia, Australia; University of California, Berkeley, Berkeley, California, USA

**Keywords:** polysaccharides, cell envelope, O antigen, peptidoglycan, Lpp, OmpA

## Abstract

Bile salts (BS) are antimicrobials that disrupt bacterial cell membranes and induce oxidative stress. The gut bacterium *Escherichia coli* is naturally resistant to BS, including the model strain K12 MG1655 that produces a lipopolysaccharide (LPS) without O-antigen (OAg) on the cell surface. Paradoxically, we have previously shown that restoring a wild-type like LPS with attached OAg (MG1655-S) sensitizes *E. coli* K12 to exogenous BS. In this study, we investigate this phenomenon. We show that mutations causing truncation of the LPS core oligosaccharide render MG1655-S strains even more susceptible to BS compared to MG1655. These mutants phenocopy a K-12 MG1655-S Δ*waaL* mutant, which is defective in OAg ligase, primarily due to periplasmic accumulation of the unligated lipid-linked UndPP-OAg. Through the characterization of BS-resistant suppressor mutants of MG1655-S Δ*waaL*, we identify key genetic disruptions involved in resistance. Notably, we observed the highest BS resistance in strains with a weaker connection between the outer membrane (OM) and peptidoglycan (PG), including strains lacking the major OM-anchored, PG-binding proteins OmpA or Lpp. Expressing versions of OmpA and Lpp that lack PG-binding capacity also enhanced the BS resistance. Our data suggest that BS-induced stress in OAg-producing *E. coli* is due to the spatial constraints between OM and PG and that mutations disrupting OM–PG interactions alleviate this stress, thereby enhancing BS resistance. These findings provide new insights into a major challenge *E. coli* faces in the gut environment, where it needs to produce OAg for stable colonization and immune evasion while resisting the antimicrobial activity of BS.

**IMPORTANCE:**

Enteric bacteria residing in the human gut must withstand host-derived antimicrobial bile salts, but resistance mechanisms are not fully elucidated. In this study, we investigate bile salt resistance mechanisms in O-antigen (OAg)-producing *Escherichia coli* K-12. We show that the accumulation of carrier lipid-linked OAg in the periplasm of strains with truncated lipopolysaccharide (LPS) core oligosaccharide or defects in OAg ligase can sensitize *E. coli* more to bile salt, unless the physical links between outer membrane and peptidoglycan are disrupted, highlighting that bile salt-induced stress is attributed to spatial constraints between the outer membrane and peptidoglycan layer. Our work uncovers a previously unappreciated envelope stress response mechanism in *E. coli*, where reducing outer membrane–peptidoglycan connectivity mitigates bile salt-induced damage arising from OAg production. These findings reshape our understanding of how physical architecture and biosynthetic intermediates intersect to influence bacterial survival in hostile environments.

## INTRODUCTION

Bile salts (BS) cholate and chenodeoxycholate are derived from cholesterol in the liver and secreted from gallbladder storage into the human small intestine. There, the two primary BS are modified by anaerobic bacteria through dehydroxylation to generate the secondary BS deoxycholate (DOC) and lithocholate, respectively (1). BS are amphipathic molecules that act as detergents to help emulsify fats, but they also possess potent antimicrobial activity by altering the permeability of the bacterial cell membrane (2, 3), unfolding cellular proteins (4), and inducing oxidative damage to DNA (5). However, enteric bacteria have adapted to the human gut and resist the antimicrobial activities of BS, a key characteristic that was exploited in the development of the selective medium MacConkey agar for the isolation and identification of gut bacteria (6). Some bacterial pathogens, including adherent-invasive *Escherichia coli* (7), *Shigella flexneri* (8), and *Salmonella enterica* (9), have even evolved to utilize BS as an environmental cue to modulate their virulence functions.

Intracellular access of toxic compounds is restricted by the outer membrane (OM) in Gram-negative bacteria, which is further fortified by effective drug efflux systems. Indeed, the major BS resistance mechanism in the model enterobacterium *E. coli* is through the exclusion of intracellular accumulation via multidrug efflux systems, such as AcrAB-TolC and MdtM (10). One might expect to be able to identify direct cellular targets of BS from an *E. coli* mutant lacking the major efflux pump TolC. In one study (11), mutations conferring BS resistance in *E. coli* K-12 Δ*tolC* were mapped to the *pstI-cyaA-crp* region, which encodes a (PTS)-cAMP-Crp regulatory cascade. These mutations reduced carbohydrate metabolism and thus the accumulation of reactive oxygen species (ROS) (12), which can damage macromolecules like DNA (13), indicating that BS induce a widely shared, oxidative stress–mediated death pathway. BS were also reported to upregulate chaperones in *Enterococcus faecalis* (14), suggesting that they induce a protein-folding stress. Indeed, BS were shown to effectively cause protein aggregation and induce disulfide stress in *E. coli* K-12 lacking the cytosolic chaperone Hsp33 (4). However, it remains unclear whether these consequences arise from a direct attack on key proteins or are downstream effects resulting from the interaction of BS with one or more unidentified target(s).

As a model enterobacterium, *E. coli* K-12 has been used frequently in studying BS resistance. However, due to a mutation in the *wbbL* gene which encodes the second glycosyltransferase for OAg biosynthesis, K-12 strains do not produce OAg (15), a virulence determinant that is present in most *E. coli* isolates and is important in colonizing the human gut (16). We have recently shown that restoring OAg production in *E. coli* K-12 sensitizes cells to the large PG-targeting antibiotic vancomycin in the presence of BS (3), an effect that was also reproduced in OAg-producing uropathogenic *E. coli* and *S. flexneri* strains, suggesting that BS induce cell-envelope stresses when OAg is produced. Prolonged exposure of the OAg-restored *E. coli* K-12 to BS selected for mutations leading to the inactivation of OAg production (3). This BS sensitization effect in OAg-producing *E. coli* was proposed to result from the accumulation of the unligated lipid-linked OAg, undecaprenol pyrophosphate-OAg (UndPP-OAg, referred to as such throughout this manuscript), since a Δ*waaL* mutant was found to be non-resistant to BS on its own when OAg is being produced but not attached to nascent LPS molecules in the periplasm (3). However, the exact cell envelope processes affected remain to be elucidated.

Here, we show that mutations leading to the truncation of LPS core oligosaccharide in OAg-producing *E. coli* K-12 render cells less resistant to BS, which can be attributed to the accumulation of UndPP-OAg in these strains. To understand the underlying mechanism of increased BS sensitivity in these *E. coli* K-12 strains accumulating UndPP-OAg, we selected and characterized suppressor mutants capable of growing in the presence of a lethal dose of BS. Suppressor mutations conferring the highest BS resistance disrupted OAg biogenesis, and those that did not were mapped primarily to genes responsible for OM biogenesis. Importantly, mutations disrupting the peptidoglycan (PG)-interacting domain of OmpA or Lpp, which both reduce the linkage between the OM and PG, conferred the highest resistance to BS. Our data suggest that BS exert stress on *E. coli* K-12 accumulating polymerized UndPP-OAg through periplasmic spatial constraint, and that the reduction in OM–PG connections confers higher levels of BS resistance.

## RESULTS

### Periplasmic accumulation of UndPP-OAg sensitizes *E. coli* to BS

**Fig 1:**
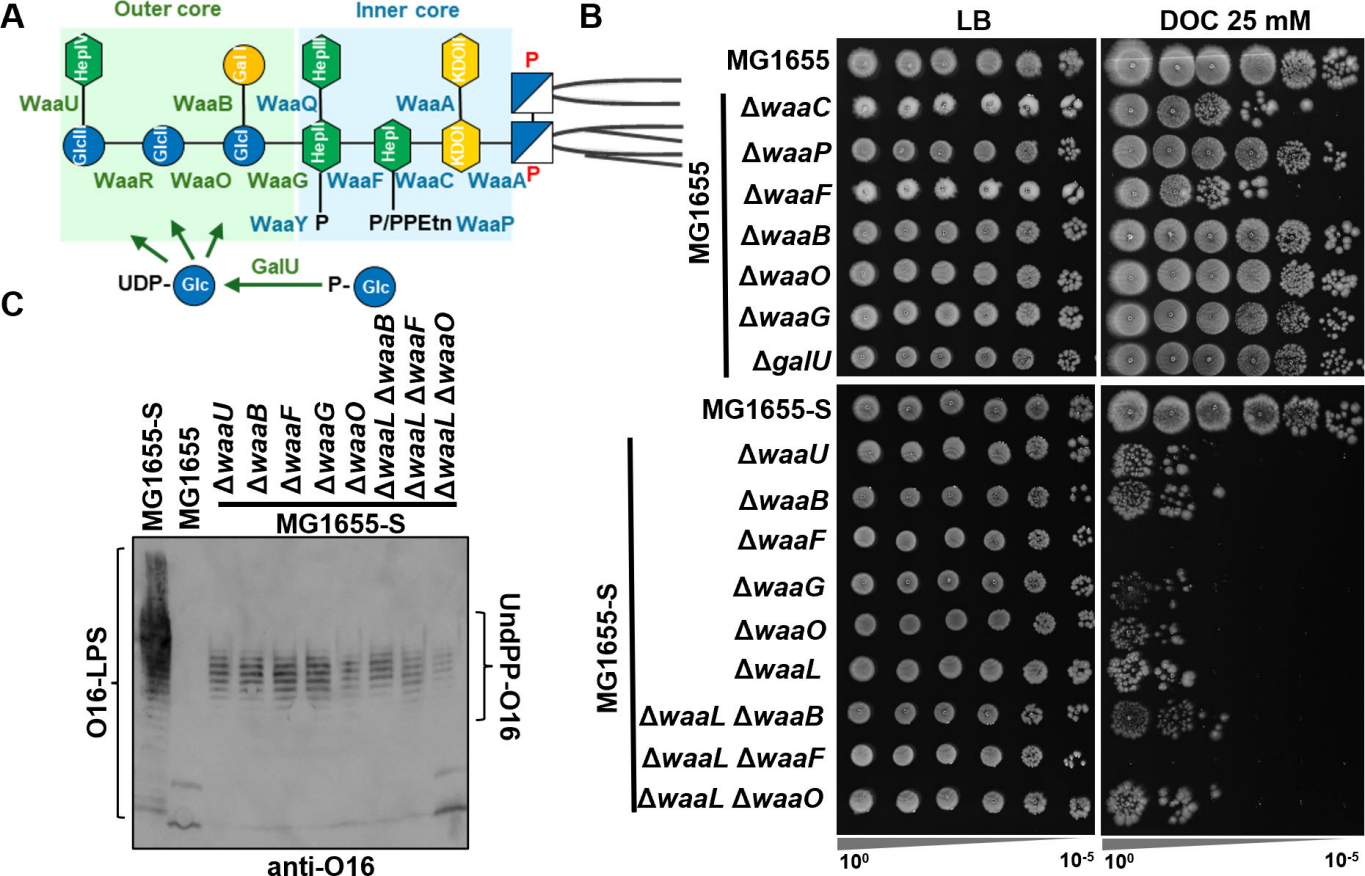
Accumulation of UndPP-OAg sensitizes *E. coli* K-12 MG1655 to BS. (**A**) Schematic of the *E. coli* K-12 LPS core oligosaccharide structure and assembly with enzymes for its assembly shown. (**B**) BS sensitivity assay of the indicated *E. coli* K-12 strains. Overnight bacterial culture was adjusted to an OD_600_ of 1 and spotted (4 µL) in 10-fold serial dilutions (10^0^–10^−5^) onto LB agar supplemented with or without 25 mM sodium deoxycholate (DOC). (**C**) Western immunoblotting of O16 OAg with lysate prepared from whole bacterial cells of indicated *E. coli* K-12 strains. Accumulated periplasmic UndPP-OAg is shown and labeled.

The bacterial OM acts as a permeability barrier primarily through LPS molecules on the outer leaflet, which limits the access of many antimicrobials to intracellular targets (17). Although LPS is essential, mutants with altered or truncated core structures remain viable. We therefore first investigated BS resistance of *E. coli* K-12 MG1655 with defects in LPS core oligosaccharide through gene deletions abolishing the inner core (Δ*waaC*, Δ*waaF*, and Δ*waaP*) and outer core (Δ*waaB*, Δ*waaO*, Δ*waaG*, and Δ*galU*) biosynthesis and assembly (Fig. 1A; Fig. S1A). Consistent with a previous report (18), only the heptose-less mutants Δ*waaF* and Δ*waaC* resulted in reduced BS resistance, and disruptions to outer core oligosaccharide and phosphorylation on heptose I (HepI) had no effect on BS resistance (Fig. 1B).

We have previously removed the *IS5* insertional element from *wbbL* in strain MG1655 to construct the OAg-producing *E. coli* K-12 strain MG1655-S (3) (Fig. S1B). Disruptions of both LPS inner core (Δ*waaF*) and outer core (Δ*waaB*, Δ*waaO*, Δ*waaG*, and Δ*waaU*) in MG1655-S resulted in truncation of LPS core oligosaccharide and loss of OAg capping due to the loss of distal HepIV required for OAg addition (Fig. S1B). All tested outer-core oligosaccharide assembly mutants in OAg-restored MG1655-S showed a drastic reduction in BS resistance (Fig. 1B), while in OAg-deficient MG1655, this sensitivity was only observed in Δ*waaF* and Δ*waaC* mutants, suggesting that the production of OAg sensitizes mutants with LPS outer-core truncation to BS. We have shown previously that an OAg ligase mutant Δ*waaL* had reduced BS resistance in MG1655-S due to accumulation of UndPP-OAg in the periplasm, but not in MG1655 (3). Here, we show that all MG1655-S core-truncation mutants accumulated UndPP-OAg (Fig. 1C), and the level of reduction in BS resistance in outer-core truncation mutants (Δ*waaB*, Δ*waaO*, Δ*waaG*, and Δ*waaU*) was similar to that of Δ*waaL* mutant in the MG1655-S background (Fig. 1B). Moreover, disruption of outer-core oligosaccharide (Δ*waaB* and Δ*waaO*) in MG1655-S Δ*waaL* did not further sensitize cells to BS compared to corresponding outer-core mutants in MG1655-S (Fig. 1B), suggesting that the sensitization to BS in these MG1655-S outer-core truncation mutants was primarily due to accumulation of UndPP-OAg. Interestingly, inner-core truncation through Δ*waaF* mutation (Fig. 1A) in both MG1655-S and MG1655-S Δ*waaL* accumulated UndPP-OAg, further inhibiting bacterial growth on the BS-containing media (Fig. 1B). This observation suggested that UndPP-OAg accumulation and the loss of heptose are additive in reducing BS resistance, likely by affecting distinct cellular pathways or independently compromising the membrane barrier.

The major known BS resistance mechanism is through removal of cellular BS via efflux pumps. We therefore examined the uptake and efflux rates of a TolC substrate ethidium bromide (EtBr) in the MG1655-S Δ*waaL* strain. In the presence of a protonophore carbonyl cyanide m-chlorophenylhydrazone (CCCP), which inhibits the function of efflux pumps through disruption of the proton motive force, the kinetics of intracellular EtBr accumulation in MG1655-S Δ*waaL* were indistinguishable from those in MG1655 and MG1655-S (Fig. S1C), suggesting that the accumulation of UndPP-OAg in MG1655-S Δ*waaL* does not affect membrane permeability. In the absence of CCCP, deletion of *tolC* in MG1655, as expected, resulted in slower EtBr efflux compared to MG1655. In contrast, MG1655-S Δ*waaL* exhibited EtBr efflux rates comparable to those of MG1655 and MG1655-S, indicating that the efflux pump activity via TolC is not impaired by the accumulation of UndPP-OAg in the periplasm of MG1655-S Δ*waaL*. Consistently, MG1655-S Δ*waaL* was not further sensitized to the TolC efflux pump substrate ampicillin (19) compared to MG1655 and MG1655-S (Fig. S1D).

### Disruptions in OAg biosynthesis strongly enhance BS resistance in MG1655-S ΔwaaL

**Fig 2:**
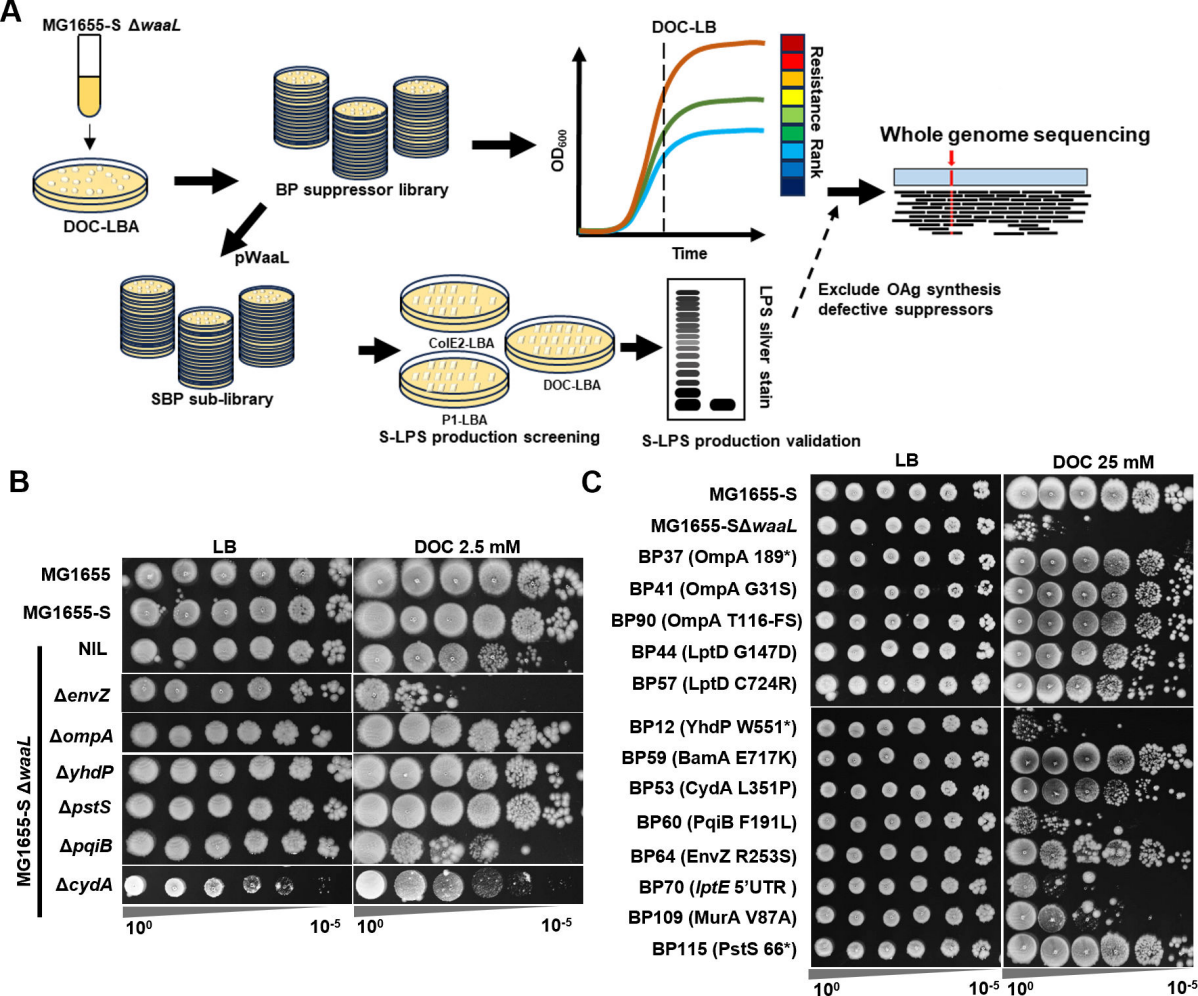
Isolation, selection, and characterization of MG1655-S Δ*waaL* BS-resistant suppressor mutants. (**A**) Schematic of suppressor mutant isolation and selection for whole-genome sequencing (see Methods and Materials for a detailed description). (**B**) BS sensitivity assay for the validation of restored BS resistance in suppressor mutations, identified by targeting non-essential genes through single-gene deletion mutants. (**C**) Selection of strong suppressor by BS sensitivity assay at an elevated concentration of BS (25 mM DOC). FS, frameshift mutation.

To investigate the underlying mechanism of BS sensitization due to the production and periplasmic accumulation of UndPP-OAg, we harvested 157 suppressor mutants of MG1655-S Δ*waaL* that grew on LB agar medium containing 2.5 mM DOC (DOC-LBA), designated as the BP suppressor library (Fig. 2A). All suppressor mutants grew on plain LB medium with no observable growth defects (Table S1). We then characterized growth kinetics of the BP library strains in liquid DOC-LB and ranked the BS resistance according to optical density at 3.5 h (Fig. 2A). A higher culture density was observed for all suppressor mutants compared to their parent strain MG1655-S Δ*waaL* (Table S1). Disruption of OAg repeating unit (RU) biosynthesis and assembly was previously shown to restore BS resistance in MG1655-S Δ*waaL* (3). We therefore first performed library screening to distinguish and exclude suppressor mutations affecting OAg biosynthesis and assembly by restoring OAg-LPS (Smooth-LPS, S-LPS) production in the full BP suppressor library via complementation with WaaL. The pWaaL-complemented BP suppressor library is designated as SBP sub-library (Fig. 2A). S-LPS production protects *E. coli* MG1655-S from colicin E2 (ColE2), DNA endonuclease cell entry, and bacteriophage P1 infection (3). We therefore patched the SBP sub-library onto agar LB medium containing ColE2 (ColE2-LBA) and P1kc phage (P1-LBA) as well as DOC-LBA (Fig. 2A). All SBP mutants retained BS resistance, growing on DOC-LBA with no observable defects, yet 14/157 SBP suppressor mutants showed growth defects on ColE2-LBA and 23/157 SBP suppressor mutants showed growth defects on P1-LBA (Table S1). Consistent with our prediction, most SBP mutants with completely inhibited growth on ColE2-LBA and/or P1-LBA corresponded to BP mutants with the highest BS resistance among all mutants. Specifically, 11 SBP mutants with defects growing on ColE2-LBA and/or P1-LBA corresponded to BP mutants ranked among the top 20 for BS resistance (Table S1). These 11 SBP mutants were further confirmed to lack detectable or altered S-LPS production (Fig. S2A) and showed compromised resistance to ColE2 (Fig. S2B). We have also performed whole-genome sequencing of two of the mutant isolates with defective S-LPS production (SBP66 and SBP73) and identified mutations in *wecA* and *wbbL* (Table 1), which encode the initial and second glycosyltransferases (IT and 2nd GT) in the assembly of OAg RU, responsible for the engaging and the committed steps of OAg RU assembly, respectively (20). These results confirmed that our screening design was sufficiently robust and accurate to successfully exclude suppressor mutations affecting OAg RU assembly and that the remaining mutants retain BS resistance despite producing OAg.

**TABLE 1:** Mutations identified in MG1655-S∆*waaL* suppressors grown in DOC-LBA

BP mutants	Nucleotide mutation and locus	Peptide mutation (codon change)	LPS in SBP
BP12	C-T substitution at 3394727	YhdP W551* (T**G**G to T**A**G)	S-LPS
BP37	G-C substitution at 1019487	OmpA Y189* (TA**C** to TA**G**)	S-LPS
BP41	C-T substitution at 1019963	OmpA G31S (**G**GT to **A**GT)	S-LPS
BP44	C-T substitution at 56607	LptD G147D (G**G**T to G**A**T)	S-LPS
BP53	T-C substitution at 772509	CydA L351P (C**T**C to C**C**C)	S-LPS
BP57	A-G substitution 54940	LptD C724R (**T**GC to **C**GC)	S-LPS
BP59	G-A substitution at 200076	BamA E717K (**G**AG to **A**AG)	S-LPS
BP60	C-A substitution at 1013831	PqiB F191L (TT**C** to TT**A**)	S-LPS
BP64	G-T substitution at 3535112	EnvZ R253S (**C**GC to **A**GC)	S-LPS
BP70	G-A substitution at 674853	5’UTR of *LptE*	S-LPS
BP90	Single A deletion at 1019706	OmpA frameshift at T116	S-LPS
BP109	A-G substitution at 3336235	MurA V87A (G**T**T to G**C**T)	S-LPS
BP115	Single C deletion at 3911347	PstS truncated to 66 AA	S-LPS
BP66	C-G substitution at 3968730	WecA P272R (C**C**A to C**G**A)	SR-LPS
BP73	T-C substitution at 2103382	WbbL Y3C (T**A**T to T**G**T)	SR-LPS

### Disruptions in OM biogenesis machinery restore BS resistance in MG1655-S ΔwaaL

In the top 31 BS-resistant BP mutants (Table S1), we excluded 11 with mutations potentially affecting OAg synthesis. The remaining 20 BP mutants were confirmed to have restored resistance to BS compared to MG1655-S Δ*waaL* (Fig. S2C). Interestingly, eight BP mutants (BP12, BP41, BP44, BP57, BP59, BP60, BP70, and BP90) lacked the elevated vancomycin resistance of MG1655-S Δ*waaL* (3) (Fig. S2C). These eight BP mutants along with another five BP mutants (BP37, BP53, BP64, BP109, and BP115), which maintained elevated vancomycin resistance (Fig. S2C), were further confirmed to have no detectable changes in S-LPS profile when complemented by WaaL in the SBP sub-library (Fig. S2D) and were subjected to whole-genome sequencing analysis (Fig. 2A).

This analysis identified nine mutations affecting different OM components and four mutations in other genes. We found mutations in OM-related genes encoding (i) OmpA (BP37, BP41, and BP90), one of the most abundant OM proteins (OMP); (ii) BamA (BP59), an essential component of the OMP biogenesis machinery; (iii) LptD (BP44 and BP57), an essential component for LPS translocation; (iv) the 5′UTR of *lptE* (BP70), encoding the lipoprotein essential for LPS assembly; and (v) YhdP and (vi) PqiB, two proteins involved in lipid transport to the OM (BP12 and BP60) (Fig. S2C; Table 1). We also identified four additional mutations in genes encoding the following: (vii) the sensor histidine kinase EnvZ (BP64), which modulates OM porins OmpC and OmpF; (viii) cytochrome bd-I ubiquinol oxidase subunit 1 CydA (BP53), which acts as a terminal oxidase producing proton motive force; (ix) UDP-N-acetylglucosamine enolpyruvyl transferase MurA (BP109), which catalyzes the first committed step in the assembly of PG; and (x) the periplasmic phosphate-binding protein PstS (BP115) (Fig. S2C; Table 1).

To investigate these suppressor mutations, we generated single-gene deletion mutants of the non-essential genes *envZ*, *ompA*, *yhdP*, *pstS*, *pqiB*, and *cydA* in the MG1655-S Δ*waaL* strain background and examined their resistance to BS. We confirmed that deletions of *ompA*, *yhdP*, and *pstS* restored BS resistance in MG1655-S Δ*waaL* (Fig. 2B), suggesting that mutational alterations identified in these genes likely resulted in loss of function (Fig. 2B). In contrast, gene deletions for *pqiB* and *envZ* did not rescue BS resistance of MG1655-S Δ*waaL* (Fig. 2B), with deletion of *envZ* instead further sensitizing MG1655-S Δ*waaL* to BS (Fig. 2B), suggesting that mutational alterations identified in these genes probably led to altered protein activity. The effect of *cydA* deletion on BS resistance of MG1655-S Δ*waaL* could not be determined, as the growth of MG1655-S Δ*waaL* Δ*cydA* in plain LBA was compromised (Fig. 2B). These results support our genotypic analysis and suggest multiple complex pathways for rescuing BS resistance of MG1655-S Δ*waaL*.

### Screening for strong suppressors

**Fig 3:**
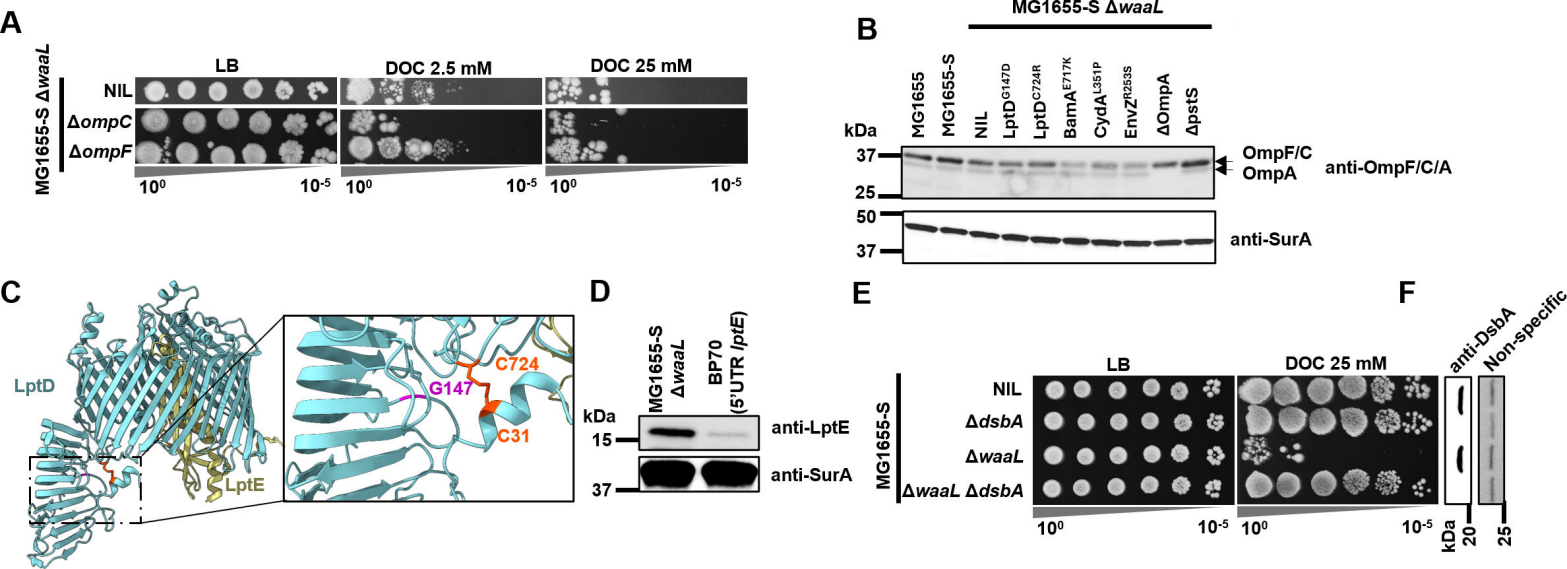
Investigation of robust MG1655-S Δ*waaL* BS suppressor mutations. (**A**) BS sensitivity assay of single-gene deletion mutants in MG1655-S Δ*waaL* with 2.5 mM or 25 mM DOC. (**B**) Western immunoblotting of OmpF, OmpC, and OmpA from whole-cell lysates of the indicated bacterial strains using anti-OmpF/C/A antibodies. Anti-SurA antibody was used to detect SurA expression as a loading control. (**C**) Identified LptD suppressor mutation mapping on the crystal structure of LptD from *Shigella flexneri* (PDB 4Q35). Mutated residues G147 and C724 are shown in magenta and orange, respectively, with the C31-C724 disulfide bridge shown. (**D**) Western immunoblotting of LptE and SurA from whole-cell lysate of the indicated bacterial strains using anti-SurA and anti-LptE primary antibodies. (**E**) BS sensitivity assay of single-gene deletion mutants in MG1655-S Δ*waaL* with 2.5 mM or 25 mM DOC. (**F**) Western immunoblotting of DsbA from whole-cell lysate of the indicated bacterial strains using anti-DsbA antibodies. A non-specific protein band detected with anti-DsbA was used as a loading control.

To further select strong suppressor mutations conferring BS resistance in MG1655-S Δ*waaL*, we challenged the 13 selected BP suppressor mutants with an elevated concentration of BS (25 mM DOC) and found that among all tested suppressors, the mutations identified in *ompA*, *lptD*, *bamA*, *cydA*, *envZ*, and *pstS* conferred the highest BS resistance to MG1655-S Δ*waaL* (Fig. 2C). An EnvZ^P41L^ mutant has previously been shown to influence BS resistance (21) through altering expression levels of OmpC and OmpF (22). OmpF was proposed to be a porin for BS OM entry (23), and OmpC was shown to be required for BS resistance (24). Indeed, deletion of *ompC* slightly further sensitized MG1655-S Δ*waaL* to BS, while deletion of *ompF* slightly increased its BS resistance (Fig. 3A). We next examined OmpC and OmpF levels in the top 13 suppressor mutants, which revealed that the mutants with BamA^E717K^ or EnvZ^R253S^ had reduced levels of OmpF/C (Fig. 3B), while other suppressor mutants had no detectable changes in the levels of OmpF/OmpC.

LptD is an essential component of the LPS transport machinery across the OM. It features a two-domain structure: a barrel that is embedded in the OM and a periplasmic domain that structurally resembles the LptA subunit. This folding enables LptD to effectively connect with periplasmic LptA to form the transenvelope bridge for LPS transport (25). We mapped two independent mutations located at the interdomain interface (Fig. 3C), both with reduced resistance to vancomycin (Fig. S2C), yet without detectable differences in S-LPS production (Fig. S2C) and ColE2 resistance (Table S1) when complemented with WaaL. Interestingly, one of the suppressor mutants, LptD^C724R^, abolishes one of the two known disulfide bridges (C31-C724) in LptD (26). Consistent with this finding, we observed that the mutation in BP70 (located in the 5′ UTR of *lptE*) led to reduced levels of LptE (Fig. 3D). This reduction was previously shown to impair native disulfide bond formation in LptD without affecting its overall production (27). Although the physiological importance of the disulfide bridge between the two domains of LptD is unclear, it was shown to be catalyzed by the periplasmic oxidative foldase DsbA (26). Consistent with the known role of DsbA in LptD disulfide bridge formation, disruption of *dsbA* in MG1655-S Δ*waaL* fully restored its BS resistance (Fig. 3E and F); however, we were unable to determine whether the restoration of BS resistance observed in MG1655-S Δ*waaL* Δ*dsbA* was solely due to the compromised disulfide bond formation in LptD.

### Characterization of *pstS* and *bamA* mutations that restore BS resistance in MG1655-S ΔwaaL

Deletion of *pstS* was previously shown to upregulate the expression of UgpB, which carries out a moonlighting function as a periplasmic chaperone that aids protein folding to confer BS resistance (28). However, in MG1655-S Δ*waaL*, (i) ectopic expression of UgpB did not restore BS resistance, (ii) disruption of *ugpB* did not further sensitize the strain to BS, and (iii) disruption of *ugpB* in the suppressor mutant MG1655-S Δ*waaL* Δ*pstS* did not re-sensitize it to BS either (Fig. S3), suggesting that the rescue of BS resistance of MG1655-S Δ*waaL* by the disruption of *pstS* is not through UgpB chaperone function.

**Fig 4:**
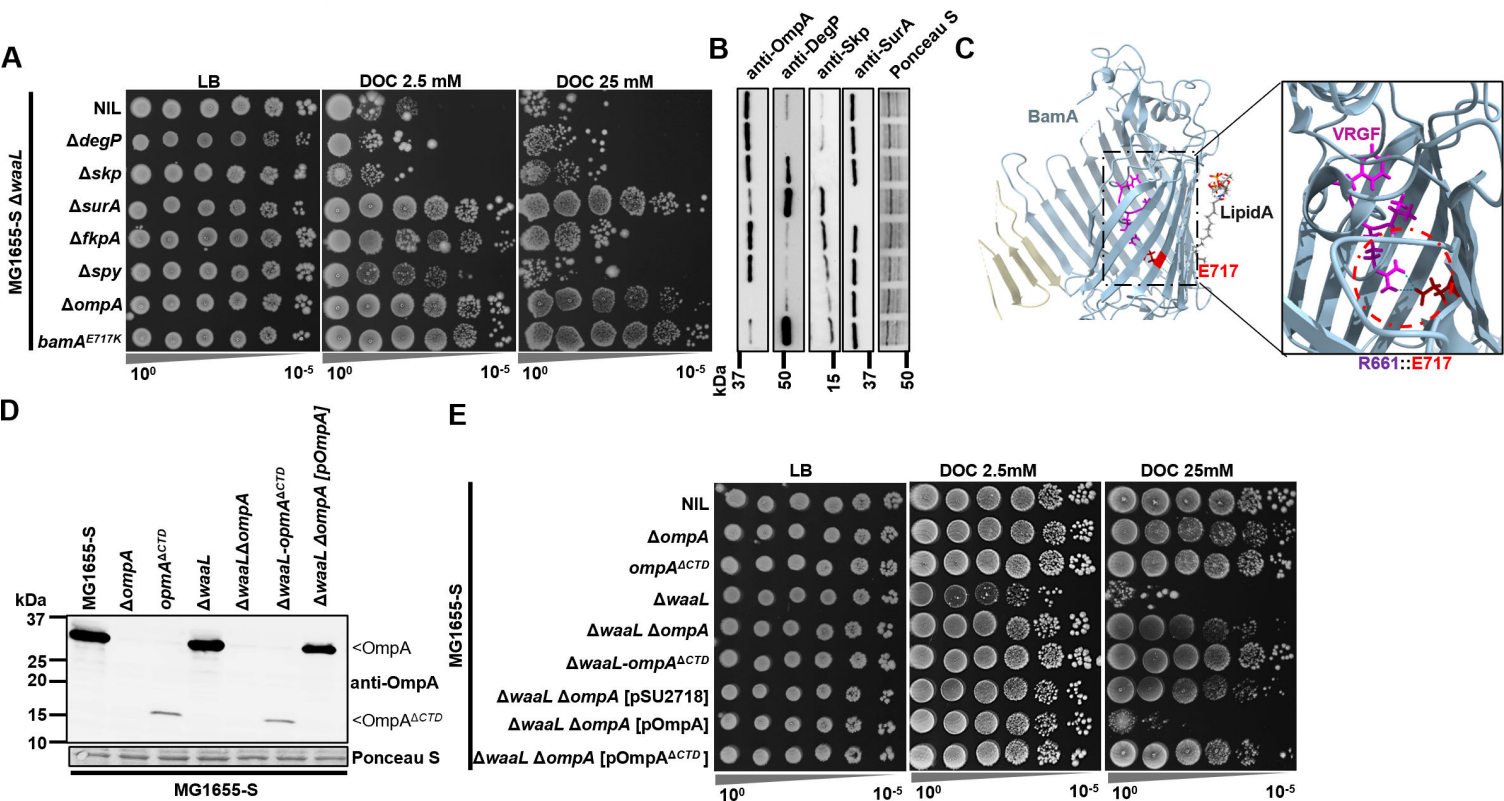
Disruption of OmpA C-terminal domain confers BS resistance to *MG1655-S* Δ*waaL*. (**A**) BS sensitivity assay of single-gene deletion mutants in MG1655-S Δ*waaL* with 2.5 mM or 25 mM DOC. (**B**) Western immunoblotting for detection of OmpA, DegP, Skp, and SurA from whole-cell lysates of indicated bacterial strains with anti-OmpA, anti-DegP, anti-Skp, and anti-SurA antibodies. Transferred proteins from cell lysate were stained with Ponceau S to indicate sample loading. Molecular weights of protein markers are indicated on the left to validate the size of the truncated OmpA protein OmpA^ΔCTD^. (**C**) BamA suppressor mutation mapping on its barrel structure (PDB 7TTC). The salt bridge between E717 and R661 in the conserved VRGF motif is shown. (**D**) Western immunoblotting for detection of OmpA from whole-cell lysates of indicated bacterial strains with anti-OmpA antibodies. Transferred proteins were stained with Ponceau S to indicate sample loading. (**E**) BS sensitivity assay of single-gene deletion mutants in MG1655-S Δ*waaL* with 2.5 mM or 25 mM DOC.

The BS DOC was reported previously as a potent protein-denaturing agent exerting disulfide stress (4), and a periplasmic chaperone FkpA was strongly upregulated in a *pstS* deletion mutant (28). We therefore investigated the role of several selected periplasmic chaperones (DegP, Skp, SurA, FkpA, and Spy) in BS resistance of MG1655-S Δ*waaL*. Interestingly, none of these chaperones alone was required in maintaining the BS resistance of MG1655-S Δ*waaL*, as their individual disruptions did not further sensitize MG1655-S Δ*waaL* to BS (Fig. 4A). Counterintuitively, disruptions of *surA*, *fkpA*, and *spy* rescued BS resistance (DOC 2.5 mM) in MG1655-S ΔwaaL, with *surA* disruption conferring the highest BS resistance (DOC 25 mM), similar to that of MG1655-S Δ*waaL* Δ*ompA* (Fig. 4A). Additionally, MG1655-S Δ*waaL* Δ*surA* was found to have reduced OmpA protein levels, with a high protein level of DegP detected (Fig. 4B), indicating a severe OM protein-folding stress.

SurA is important in OMP folding as it delivers its OMP clients to BamA for folding into the OM (29). In the suppressor mutant BP59, the mutation E717K was mapped on the OMP foldase BamA of the BAM foldase structure (Table 1). BamA E717 forms salt bridges with R661 in BamA, which is important in stabilizing a conserved VRGF motif (Fig. 4C) (30). Disruptions of the VRGF motif were shown previously to compromise BamA folding activity, leading to reduced levels of OMPs, including OmpF, OmpC, and OmpA (30). Indeed, the BamA^E717K^ suppressor mutant in the MG1655-S Δ*waaL* background also showed reduced levels of OmpF, OmpC (Fig. 3B), and OmpA (Fig. 4B). Intriguingly, in the 13 selected suppressors conferring top-ranked BS resistance to MG1655-S Δ*waaL*, three independent mutations were mapped in *ompA*, and these conferred the highest BS resistance (Table 1). Additionally, the BamA^E717K^ mutation and *surA* deletion strains were also found to have reduced OmpA levels conferring high BS resistance to MG1655-S Δ*waaL*. However, it was unable to determine whether the rescue effect was due to reduced OmpA level alone, since the folding of LptD also requires BamA (31) and SurA (32). Together, these results suggest that the biogenesis of OmpA induces stress in MG1655-S Δ*waaL* in the presence of BS, and we therefore focused our further studies on *ompA* mutations.

### Disruptions of OmpA CTD restore BS resistance in MG1655-S ΔwaaL

OmpA is predicted to be a two-domain protein with an N-terminal domain (NTD, aa 22-192) forming an 8-strand beta-barrel embedded in the OM, and a C-terminal domain (CTD, aa 193-346) folded independently in the periplasm. Interestingly, the mutation found in the BP37 mutant (Y189*) would truncate the CTD of OmpA but allow its barrel to be produced. We therefore generated chromosomal deletions of OmpA CTD (aa Δ190-346, designated as OmpA^ΔCTD^) in both MG1655-S and MG1655-S Δ*waaL* and confirmed the truncation of OmpA, which was detected at reduced levels (Fig. 4D). While both *ompA* and *omp*^Δ^*^CTD^* had no detectable effect on cell survival of MG1655-S in BS containing media (Fig. 4E), these mutations increased BS resistance in MG1655-S Δ*waaL*. Intriguingly, *omp*^Δ^*^CTD^* conferred greater BS resistance compared to Δ*ompA* in MG1655-S Δ*waaL* (Fig. 4E). This was further confirmed via complementation experiment. Complementation of MG1655-S Δ*waaL* Δ*ompA* mutant with WT *ompA* re-sensitized the strain to BS, but complementation with *ompA*^ΔCTD^ did not (Fig. 4E). Together, these results strongly suggest that production of the OmpA barrel is beneficial to BS resistance, while the production of OmpA with its CTD is detrimental to the survival of MG1655-S Δ*waaL* in the presence of BS.

### OM–PG anchoring is detrimental to BS resistance in MG1655-S ΔwaaL

OmpA CTD was previously shown to be involved in the regulation of Rcs stress response through interacting with RcsF (33). However, we found that the disruption of *rcsF* had no effect in rescuing the BS resistance in MG1655-S Δ*waaL* (Fig. S4). The periplasmic CTD of OmpA adopts a globular structure that is highly similar to known PG-binding domains of RmpM (*Neisseria meningitidis*) and MotB (*Helicobacter pylori*) (34) and has been proposed to interact with PG (35). Therefore, it is possible that OM tethering through interaction between OmpA and PG sensitizes MG1655-S Δ*waaL* to BS.

**Fig 5:**
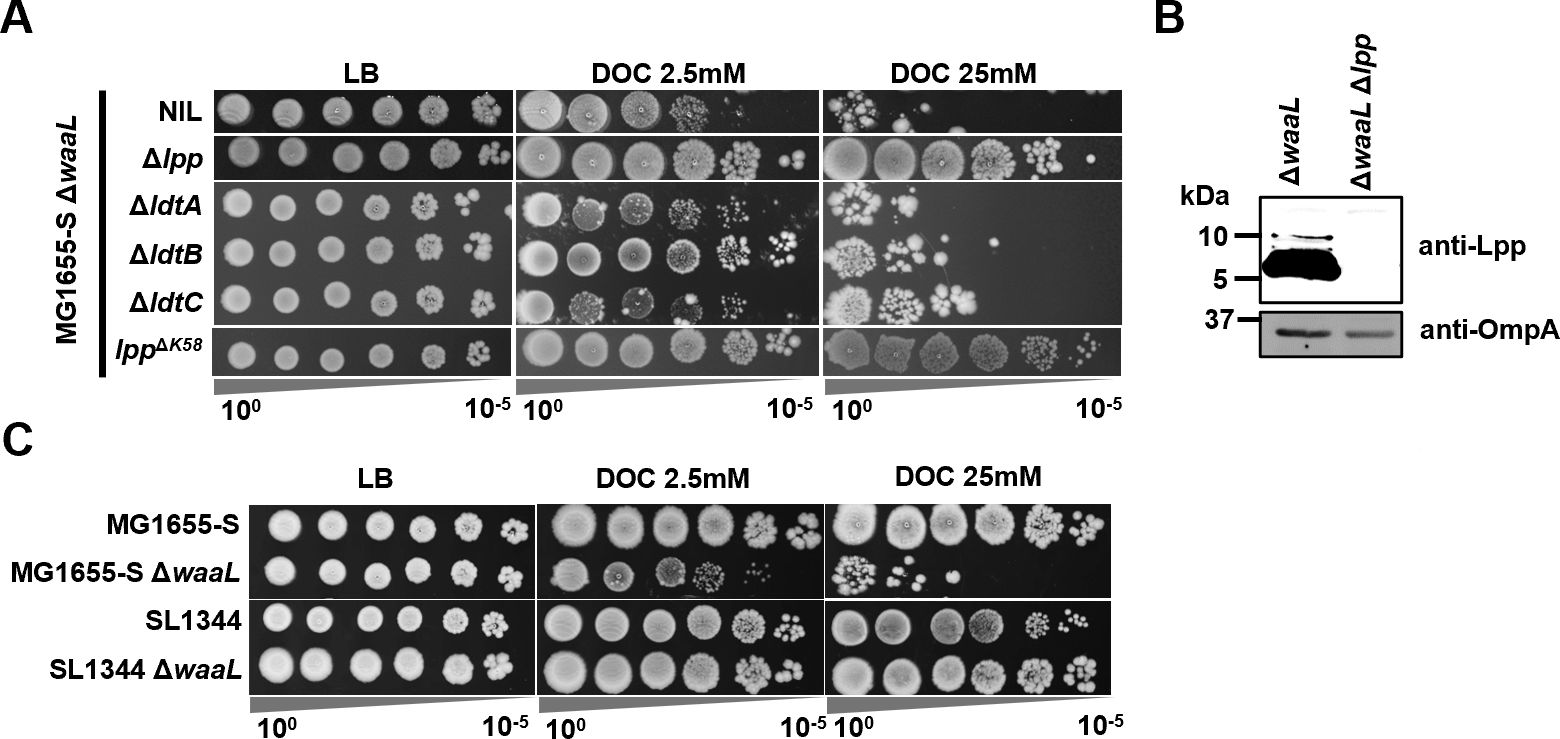
Disruption of Lpp and PG linkage confers BS resistance to *MG1655-S* Δ*waaL*. (**A**) BS sensitivity assay of single-gene deletion mutants and Lpp protein mutant in MG1655-S Δ*waaL* with 2.5 mM or 25 mM DOC. (**B**) Western immunoblotting of Lpp from whole-cell lysates of indicated bacterial strains with anti-Lpp antibodies. OmpA was detected with anti-OmpA antibodies to serve as a loading control. Molecular weight is indicated. (**C**) BS sensitivity assay indicated strains with 2.5 mM or 25 mM DOC.

To test this hypothesis, we first examined the Pal-TolA transenvelope system, which facilitates OM–PG interactions non-covalently via the PG-associated lipoprotein Pal (36). However, disruption of *pal* or *tolA* in MG1655-S Δ*waaL* further increased sensitivity to BS (Fig. S7), suggesting that disruption of a more stable or abundant OM–PG tethering mechanism might be necessary to confer BS resistance. We then investigated another, more abundant protein that tethers the OM and PG, Braun’s lipoprotein (Lpp). Lpp is covalently linked to PG by three redundant LD-transpeptidases, LdtA, LdtB (catalyzes majority of the Lpp–PG attachment), and LdtC, between the Lpp^K58^ and PG^mDAP^ residues (37). We therefore deleted *lpp* in MG1655-S Δ*waaL* and found that, surprisingly, the disruption of *lpp* fully restored BS resistance of MG1655-S Δ*waaL* (Fig. 5A and B). We verified that the restoration of BS resistance was due to the Lpp–PG linkage, since disruptions of *ltdA*, *ltdB*, or *ltdC* all marginally restored BS resistance (Fig. 5A) and the point mutant of Lpp^ΔK58^ fully restored BS resistance (Fig. 5A). The linkage between Lpp and PG is lethal when lipoprotein transport is impaired, leading to the PG attachment of IM-accumulated Lpp (38). To investigate whether BS affected lipoprotein transport in MG1655-S Δ*waaL*, we fractionated membranes of MG1655, MG1655-S, and MG1655-S Δ*waaL* grown in the presence and absence of BS (Fig. S5A) and confirmed the absence of Lpp in the IM fractions and the successful separation of IM and OM of all strains growing in the presence of BS (Fig. S5B). These results suggest that lipoprotein transport was not affected in MG1655-S Δ*waaL* when growing in the presence of BS. Additionally, we also confirmed that UndPP-OAg still accumulated when growing in the presence or absence of BS for all strong suppressor mutants reported in this work, including MG1655-S Δ*waaL* Δ*lpp* (Fig. S6), indicating that the restoration of BS resistance in these strong suppressor mutants was not due to the loss of UndPP-OAg accumulation. Taken together, these results suggest that abundant OM–PG tethering renders MG1655-S Δ*waaL* unfit for growth in the presence of BS.

BS was shown previously to induce PG remodeling with reduced level of Lpp–PG linkage for *Salmonella enterica* SL1344 (39). However, MG1655-S Δ*waaL* growing in the presence and absence of BS (Table S3; Fig. S8) resulted in similar levels of Lpp–PG crosslinking. Interestingly, unlike MG1655-S Δ*waaL*, *S. enterica* SL1344 Δ*waaL* maintained the resistance to BS (Fig. 5C). These results further support that loss of crosslinking between OM and PG confers BS resistance.

## DISCUSSION

### Accumulation of UndPP-OAg is a major contributor to the sensitivity to BS

In this study, we compared the BS sensitivity across a set of isogenic *E. coli* K-12 mutants capable of assembling OAg in the periplasm but unable to ligate it to nascent LPS molecules precluding cell-surface OAg localization. These analyses revealed that core-oligosaccharide truncation mutants accumulated UndPP-OAg, showing elevated BS sensitivity compared to their corresponding OAg-defective strains. This work strongly supports our model that the presence and accumulation of polymerized UndPP-OAg in the periplasm pose stresses in the presence of BS (3).

### Weakened LptD interdomain interactions confer high BS resistance.

**Fig 6:**
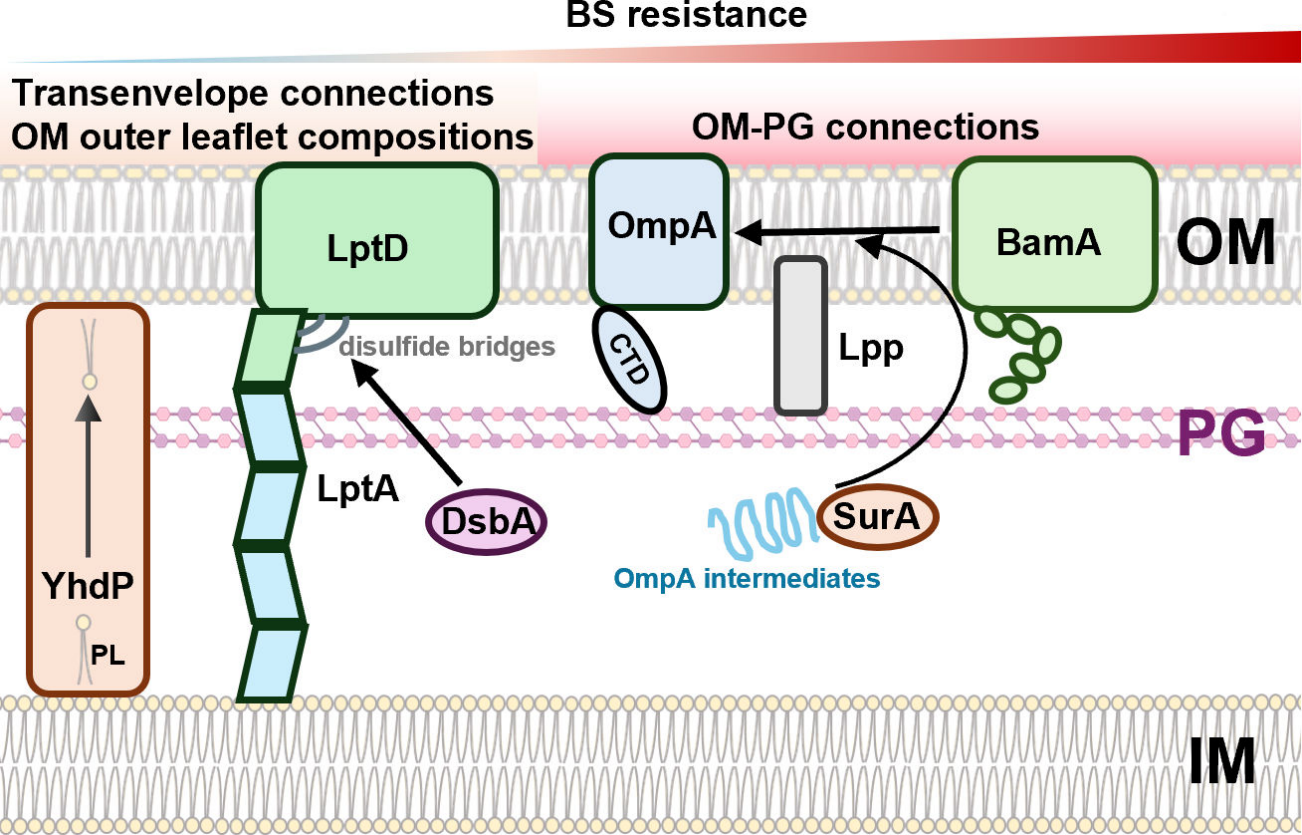
Summary of major cell envelope suppressor mutants with restored BS resistance in MG1655-S Δ*waaL*. UndPP-OAg poses cell envelope stresses in the presence of BS, presumably by affecting PG biosynthesis. Disruptions in YhdP, which is responsible for phospholipid (PL) transport, confer weak BS resistance. Disruptions in LptD interdomain interactions, OM–PG connecting molecules OmpA and Lpp, OMP biogenesis components BamA and SurA, and the cell envelope oxidase/foldase DsbA confer strong BS resistance.

At the elevated concentration of BS, we identified two independent mutants with suppressor mutations mapped in *lptD*, encoding the two-domain OM protein responsible for the translocation of LPS molecules (Fig. 6). We identified, in addition to *dsbA*, the suppressor mutation in *cydA* also fully rescued the BS resistance of MG1655-S Δ*waaL*, similar to the *lptD* suppressor mutation (LptD^C724R^) affecting the interdomain disulfide bond formation. CydA is a subunit of cytochrome *bd*, a quinol oxidase that participates in the energy metabolism of bacterial cells, providing the oxidizing power which fuels the DsbA–DsbB–ubiquinone complex for the catalysis of disulfide bridge formation during folding periplasmic proteins (40). It is likely that the suppressor mutations affecting CydA also indirectly affect LptD interdomain disulfide bond formation. The other suppressor mutation (LptD^G147D^) was also found in the interdomain interface. These two LptD suppressor mutants all exhibited reduced levels of vancomycin resistance compared to MG1655-S Δ*waaL*, again suggesting that the rescue was not through excluding BS via improved OM barrier function. Additionally, when complemented with pWaaL, these two mutants were able to produce S-LPS at a level indistinguishable from pWaaL-complemented MG1655-S Δ*waaL* via LPS silver staining and colicin E2 sensitivity assay, indicating that the production and translocation of S-LPS are not appreciably affected. It thus remains unclear what role of LptD was affected when interdomain interaction is altered through these two suppressor mutations. Nevertheless, our data tempt us to suggest that altered/weakened LptD interdomain interaction could confer MG1655-S Δ*waaL* BS resistance.

### Weakened interactions between OM and PG confer high BS resistance.

Our data suggest a model in which normal-level OM tethering to the PG layer reduces the fitness of cells exposed to BS and accumulating UndPP-OAg in the periplasm. This is consistent with a previous report in *S. enterica*, where disruptions of transpeptidases catalyzing the covalent attachment of Lpp to PG resulted in hyper-resistance to BS (39). However, unlike *E. coli* K-12 MG1655-S Δ*waaL*, *S. enterica* SL1344 Δ*waaL* was found to be fully resistant to BS. In line with our model, this could be explained by a reduced level of Lpp–PG linkages that were reported for *S. enterica* cells growing in the presence of BS (39), whereas MG1655-S Δ*waaL* showed similar levels of Lpp–PG linkages in the presence and absence of BS. The drastic differences may be accounted for by the unique human niche of *S. enterica* compared to other BS-resistant enteric bacteria, as the species exhibits a striking ability to withstand exceptionally high concentrations of BS (>60%), enabling its survival in the gallbladder, the storage site for bile (41). Given our findings, this could entail that *S. enterica* might be able to remodel its Lpp–PG linkages in response to BS, an adaptive regulatory mechanism that appears to be absent in *E. coli* K-12. Hence, *Salmonella* spp.’s ability to alter the degree of Lpp–PG crosslinking in response to BS exposure might contribute to its adaptation to host niches with high bile concentrations, setting them apart from other enteric bacteria.

Disruption of OM–PG linkages through gene deletions of *lpp* and *ompA* is known to cause OM shedding via the formation of outer membrane vesicles (OMVs) (42). While excess OMV shedding may act as a reservoir to sequester high levels of BS, thereby mitigating its toxic effects, three distinct lines of evidence argue against this idea: (i) mutants lacking *pal* or *tolA*, components of another OM–PG interacting system also known to increase OMV production upon disruption (43)*,* further sensitized MG1655-S Δ*waaL* to BS; (ii) both *lpp* and *ompA* suppressor phenotypes were robust even at low cell densities on agar plates (i.e., 10^5^-fold dilutions), where minimal OMV concentrations would be insufficient to neutralize the bulk BS in the surrounding medium; and (iii) in contrast to the robust growth observed on solid medium, all suppressor mutants, regardless of their OMV production, displayed mild lysis in liquid medium, albeit less severe than MG1655-S Δ*waaL*. This suggests that their cell envelope integrity remains compromised, independent of potential OMV effects. These results collectively suggest that OMV shedding is not the primary mechanism of BS resistance in the suppressor mutants of MG1655-S Δ*waaL*. Although deletion of *tolA* increased BS sensitivity in our study and in a previous report (44), naturally occurring intragenic tandem repeat variations in the coding region of *tolA* have been shown to modulate BS resistance. Specifically, a higher number of tandem repeats, resulting in an extended coiled-coil domain in the periplasmic region of TolA, correlates with increased BS resistance (44). This extension may enhance OM flexibility and increase membrane fluidity, contributing to resistance. This seems to agree with the data generated from OmpA suppressor mutants. A recent study reported that the connection between OM to PG mediated by OmpA was found to maintain tensile strength and OM stiffness (45). This predicts that the loss of OmpA would increase the fluidity of the OM. Collectively, these lines of evidence suggest that loss/weakening of interactions between OM and PG may decrease membrane stiffness when OAg is produced, conferring a benefit to cells in adapting to high concentrations of BS.

In conclusion, this study provides compelling evidence that a reduction in the strength of OM–PG interactions in the OAg-producing bacteria significantly enhances resistance to BS. This strategy likely enables enteric bacteria to adapt to host niches where BS are present. Our findings highlight the critical role of cell envelope plasticity in bacterial adaptation to challenging host environments.

## MATERIALS AND METHODS

### Bacterial strains and plasmids

The bacterial strains and plasmids used in this work are listed in Table S2. Single colonies of bacterial strains grown overnight on Lysogeny Broth (LB)-Lennox (46) agar (1.5% [wt/vol]) plates were picked and grown overnight in LB at 37°C for subsequent experiments. Where appropriate, media were supplemented with ampicillin (Amp, 100 µg/mL), kanamycin (Kan, 50 µg/mL), chloramphenicol (Chl, 25 µg/mL), vancomycin (Van, 200 µg/mL), sodium deoxycholate (DOC, 2.5 mM or 25 mM), purified Colicin E2 DNA endonuclease (ColE2, 100 µg/mL) (3), anhydrotetracycline (AhTet 50 ng/mL), or arabinose (Ara, 10 mM).

### Bacterial phage and phage lysate preparation

Bacteriophage preparation was done as described previously (3). Briefly, bacteriophage P1kc (ATCC 11303-B23) lysate was prepared by combining 100 µL of mid-exponential culture of MG1655 with phage stocks at a multiplicity of infection of approximately 0.5 in 3 mL of LB soft agar (0.75% [wt/vol] agar) supplemented with MC salts (100 mM MgSO_4_ and 5 mM CaCl_2_). This mixture was then poured onto LB agar plates and incubated at 37°C for 18 h. The clear upper layer of soft agar containing the phage lysate was carefully scraped off, mixed with 2 mL of LB-Miller media (LB supplemented with MC salts), vortexed, and centrifuged. The resulting clear supernatant, which contained the bacteriophage, was collected and sterilized by adding 10 µL of chloroform. Phage titers were determined by spotting 5 µL of tenfold serially diluted phage stock onto top LB soft agar plates inoculated with 100 µL of MG1655 mid-exponential culture. Plaque-forming units (pfu) were counted after incubating the infected plates at 37°C for 18 h.

### Plasmid construction

For generation of expression constructs, the coding sequences of targeted proteins were PCR-amplified from genomic DNA prepared using Qiagen QIAamp DNA Blood Mini Kit, according to the manufacturer’s protocol, and cloned into indicated plasmids via restriction enzyme cloning (NEB).

### Bacterial mutagenesis via allelic exchange

Mutagenesis was conducted following previously established protocols (47) with laboratory-optimized modifications (48). Briefly, bacterial strains containing the plasmid pKD46 were cultured overnight in 10 mL of LB broth at 30°C and subsequently sub-cultured at a ratio of 1:100 into 10 mL of LB in a 50 mL tube. Induction of lambda phage-derived protein expression was achieved by adding 50 mM L-arabinose when the optical density at 600 nm (OD_600_) reached 0.3, followed by a 1-h incubation. The bacterial cells were harvested through centrifugation (5,000 × *g*, 5 min), washed twice with 10 mL of ice-cold water, and resuspended in 100 µL of 10% (vol/vol) ice-cold glycerol for electroporation. The *cat* or *neo* gene was amplified by PCR from pKD3 or pKD4, respectively, using primers that included 40–50 bp of homologous sequences flanking the target gene (Table S2). The purified PCR amplicon (1.5 µg) was introduced into electrocompetent cells via electroporation, and the cells were immediately allowed to recover in 3 mL of LB in a 50 mL Falcon tube for 2 h at 37°C before plating 100 µL onto LB agar plates supplemented with chloramphenicol (Chl) or kanamycin (Kan). The plates were then incubated at 37°C for 16 h to facilitate the selection of mutants. Successful mutants were subsequently screened and confirmed by PCR.

All mutagenesis, including both knockouts and knocking-ins, was performed using lambda red mutagenesis, and the primers are listed in Table S2 with clear annotations. For the OmpA^Δ*CTD*^ chromosomal mutation, the OmpA CDS of 190–346 was deleted, and the reverse knockout primer was engineered to include a His_6_ tag coding sequence and a stop codon. For the Lpp^Δ*K58*^ mutation, the codon of K58 was deleted with forward primers including a stop codon after R57.

### Antibiotic susceptibility testing

Ampicillin minimum inhibitory concentrations were determined for bacterial strains according to the Clinical and Laboratory Standards Institute guideline (49), using ampicillin MIC test strips (Liofilchem, 920031), according to manufacturer’s protocol.

### Ethidium bromide accumulation and efflux assay

Ethidium bromide (EtBr) accumulation and efflux assay were performed as previously described (50). Briefly, bacterial cells grown to exponential phase (OD_600_ of 0.6) were harvested and washed with PBS. For EtBr accumulation assay, cells were resuspended in PBS (adjusted to OD_600_ of 0.2) supplemented with 1 µg/mL EtBr and 20 µg/mL carbonyl cyanide m-chlorophenylhydrazone (CCCP) and dispensed in a microtiter plate (150 µL). Intracellular EtBr accumulation was monitored in a CLOARIOstar plate reader (BMG, Australia) with excitation of 525  ±  15 nm and emission of 615  ±  20 nm at 25°C for 1 h. For EtBr accumulation, following 1-h EtBr accumulation described above, cell suspension was washed three times with PBS, resuspended in PBS supplemented with 0.4% (wt/vol) glucose, dispensed in a microtiter plate (150 µL), and fluorescence intensity was monitored as above at 37°C for 30 min. All experiments were performed in three biological replicates.

### Bacterial BS and vancomycin sensitivity assay

Bacterial survival spotting assay was performed as described previously (3). Briefly, overnight bacterial cultures were adjusted to an OD_600_ of 1 and serially diluted 10-fold to 10^−7^ with fresh LB medium. For each dilution preparation, 4 µL were spotted onto LB agar plates with indicated supplements, where appropriate.

### Bacterial growth assay

Bacterial growth kinetics were recorded as previously outlined (3). Briefly, overnight bacterial cultures were diluted 1:200 into 200 µL of fresh LB medium, with indicated supplements where appropriate, in a 96-well plate. The plates were incubated at 37°C with aeration in a CLARIOstar plate reader (BMG, Australia), which was set to measure the OD_600_ every 6 min for a duration of 18 h.

### LPS silver staining

LPS silver staining was conducted following previously established methods (3). Briefly, bacterial cells (10^9^ CFU) harvested during mid-exponential growth were collected by centrifugation (20,000 × *g* for 1 min) and lysed in 50 µL of SDS sample buffer, followed by heating at 100°C for 10 min. The samples were then cooled to room temperature and treated with 50 µg/mL proteinase K (PK, NEB) for 18 h at 60°C. After this treatment, the samples were heated again at 100°C for 10 min, and 2–5 μL of each sample was loaded onto 10–20% SDS–tricine gels (Invitrogen, #EC66252BOX), with LPS being silver stained as previously described (51).

### Colicin E2 DNA endonuclease sensitivity assay

Determination of colicin E2 DNA endonuclease sensitivity was carried out as previously described (3). Briefly, overnight bacterial culture was adjusted to an OD_600_ of 0.5 and spread onto an LB agar plate using a sterile cotton tip applicator. Plates were allowed to dry at room temperature, and 5 µL of purified colicin E2 (3) diluted in 2-fold series in PBS was spotted onto the plate. Plates were incubated overnight at 37°C for 18 h, and the sensitivity level was determined by the minimum colicin E2 concentration that showed clear bacterial growth inhibition.

### MG1655-S ΔwaaL BS suppressor mutant (BP) library acquisition and profiling

Suppressor mutants of MG1655-S Δ*waaL* resistant to DOC were obtained by spreading an overnight culture of MG1655-S Δ*waaL*, grown in LB (diluted 1:2,000 in fresh LB media), onto LB agar plates supplemented with 2.5 mM DOC. The plates were then incubated overnight at 37°C. A total of 157 independent suppressor mutants (designated as BP library) were isolated and carefully streaked onto non-selective LB plates, which were confirmed with no observable growth defects and subsequently stored at −80°C for further analysis.

To rank the DOC resistance levels of the MG1655-S Δ*waaL* suppressor mutants in the BP library, overnight cultures of the bacterial strains, grown in LB medium at 37°C without any observable growth defects, were diluted 1:100 in 200 µL of LB medium supplemented with 2.5 mM DOC in 96-well plates. These plates were incubated at 37°C for 6 h, and the OD_600_ was recorded every 6 min as described above. Mutants were ranked according to OD_600_ readings at the 3.5-h mark, with higher OD_600_ values indicating greater resistance to DOC.

### Construction of waaL-complemented suppressor mutant (SBP) sub-library and selection

To exclude the suppressor mutants with mutations abolishing OAg biosynthesis and assembly, the primary suppressor library was complemented by pWaaL (3) via polyethyleneglycol (PEG) chemical transformation (52). Briefly, MG1655-S Δ*waaL* suppressor mutants were grown to an OD_600_ of 0.2 in 1 mL LB media and were harvested via centrifugation (5,000 × *g*, 4°C). Bacterial cell pellets were mixed with 100 µL of ice-cold TSS buffer (LB medium, 10% [wt/vol] PEG [MW ~3,500 Da], 10 mM MgCl_2_ and 10 mM MgSO_4_, pH 6.5) supplemented with 1 ng of pWaaL and incubated on ice for 30 min. After incubation, 900 µL of LB supplemented with 0.2% glucose was added, and the reaction was further incubated at 37°C with shaking for 1 h before plating on LB agar plates supplemented with appropriate antibiotic. Plates were incubated for 18 h at 37°C to select for transformants. All 158 MG1655-S Δ*waaL* suppressor mutants yielded transformants which were stored and designated as SBP library.

To identify mutants that potentially harbor suppressor mutations abolishing OAg biosynthesis and assembly, strains from the SBP library were patched onto LB agar plates supplemented with 100 µg of ColE2, 2.5 mM DOC, or LB agar plates pre-spread with 100 µL of P1kc phage lysate at 10^11^ pfu, respectively. Plates were incubated at 37°C to observe sensitivity to ColE2, DOC, and P1kc phage. The corresponding SBP mutants of top-ranked (top 31) BP suppressor mutants, according to DOC resistance, were further validated via LPS silver staining and ColE2 sensitivity assay, as detailed above. Suppressor mutants with identified defects in OAg biogenesis were excluded for whole-genome sequencing analysis.

### Whole-genome sequencing

For whole-genome sequencing of bacterial strains, genomic DNA from MG1655-S Δ*waaL* and suppressor mutants was extracted using the Qiagen QIAamp DNA Blood Mini Kit, according to the manufacturer’s instructions. Samples were then prepared for DNBseq DNA library construction (BGI) and subsequently sequenced using DNBSEQ PE150 technology (BGI). The processed reads for each strain were mapped to the NCBI MG1655 reference genome (Accession number U00096) using Geneious 8.0. Sequence variations between our laboratory strain of MG1655 and the online reference genome, determined and reported previously (3), were excluded.

### Western immunoblotting

For Western immunoblotting, whole bacterial cell lysates (10^9^ cells) were prepared in 100 µL SDS sample buffer and heated at 95°C for 5 min. Protein was separated via SDS-PAGE and transferred (2.5A, 6 min) onto nitrocellulose membrane and detected with antibodies. Successful protein transfer was confirmed with ponceau S staining and imaged to serve as a loading control. For Western immunoblotting of O16 LPS, polysaccharide samples were separated by SDS-tricine gel electrophoresis and processed as above. Rabbit polyclonal anti-O16 antibodies were purchased from SSI Diagnostica (#SSI85012). Rabbit polyclonal anti-OmpA is a generous gift from Prof. Ulf Henning (Max Planck Institute for Biology Tübingen). Rabbit polyclonal anti-SurA, rabbit polyclonal anti-Skp, and rabbit polyclonal anti-LptE are generous gifts from Prof. Thomas Silhavy (Princeton University). Rabbit polyclonal anti-MBP-DegP is a generous gift from Prof. Michael Ehrmann (University of Duisburg-Essen). Rabbit anti-AcrB is gifted by Prof. Reiter Venter (University of South Australia). Anti-Lpp antibody was affinity purified from anti-OmpF/C/A antibodies that are generous gifts from Prof. Rajeev Misra (Arizona State University). Briefly, anti-OmpF/C/A antibody (1/100 in PBS) was incubated overnight at 4°C with nitrocellulose membrane area containing Lpp protein transferred from whole-cell lysate. Membranes were washed three times with PBS containing 0.5% (vol/vol) Tween 20 and eluted with 100 µL of 0.1 M glycine, pH 3.0. Eluted antibodies were pH adjusted to pH 7.0 and verified with Western immunoblotting.

### Protein sequence alignment and structure comparison analysis

The BamA structure (PBD 7TTC) (53) and LptD structure (PDB 4Q35) (54) were analyzed and annotated by using UCSF Chimera X (55).

### Membrane fractionation by sucrose density gradient centrifugation

Bacterial cell membrane fractionation was performed as described previously (56) with modifications. Briefly, overnight bacterial culture was subcultured 1:100 in 100 mL LB and grown for 2 h at 37°C until OD_600_ reached 0.8. DOC was added to 0.5 mM and allowed to grow for another 1 h. Cells were harvested via centrifugation at 5,000 × *g*, and the resulting cell pellet was resuspended in 5 mL osmotic buffer (0.5 M sucrose, 10 mM Tris, pH 7.5), treated with lysozyme (200 µg/mL) and 1 mM EDTA on ice for 10 min and sonicated to lyse cells (with approx. 1,000 J accumulative energy). Sonicates were briefly centrifuged at 5,000 × *g* for 1 min at 4°C, and the resulting supernatant was sedimented via ultracentrifuge at 140,000 × *g* for 1.5 h (Beckman Coulter type 70.1 rotor with tube #355603). The resulting membrane pellet was then dissolved in 1 mL low-density isopycnic sucrose gradient solution (20% [wt/vol] sucrose, 1 mM EDTA, 1 mM Tris, pH 7.5). Centrifuge tubes were filled with 2 mL of (73% [wt/vol] sucrose, 1 mM EDTA, 1 mM Tris, pH 7.5), 4 mL of (45% [wt/vol] sucrose, 1 mM EDTA, 1 mM Tris, pH 7.5), 1 mL of solubilized membrane fraction, and topped up with ~6 mL of (20% [wt/vol] sucrose, 1 mM EDTA, 1 mM Tris, pH 7.5) until tubes were full. Centrifugation was performed at 100,000 *× g* for 17 h at 4°C (Beckman Coulter SW40 Ti) with slow acceleration and no brake. Tubes containing separated membranes were imaged. Fractions (1 mL) were collected by 1 mL P1000 tip (with tip cut by ~5 mm). For the NADH dehydrogenase assay, each isolated fraction was diluted 1:10 in 10 mM Tris (pH 7) buffer (10 μL + 90 µL) in a 96-well plate, followed by the addition of 10 µL of 10 mg/mL NADH (in MQ water). Plates were immediately read at 340 nm every 30 s for 40 min. Readings at 20 min were compared, with low optical density at 340 nm, indicating high enzyme activity of NADH dehydrogenase. All fractions were subjected to Western immunoblotting as described above and detected with anti-OmpA, anti-AcrB, and anti-Lpp antibodies.

### Peptidoglycan purification and quantification of muropeptides

Peptidoglycan isolation and purification were performed as described previously (57). Briefly, a 400 mL bacterial cell culture grown to an OD_600_ of 0.8 was supplemented with 2.5 mM DOC for 30 min, rapidly cooled in an ice–water bath, and then centrifuged (10,000 × *g*, 10 min, 4°C). Cell pellets were resuspended in 6 mL of ice-cold water and dropped into boiling 8% (wt/vol) SDS solution. The samples continued to be boiled for 30 min before being subjected to ultracentrifugation (437,000 *× g*, 1 h), with repeated washes with water to remove SDS. The SDS-free pellet was resuspended in 10 mM Tris-HCl (pH 7.0) containing 320 mM imidazole and incubated with 150 µg/mLα-amylase (Sigma) at 37°C for 2 h, followed by incubation with 200 µg/mL preheated pronase E (Sigma) at 60°C for 2 h. The sample was then mixed with an equal part of 4% SDS and boiled for 30 min. PG was then harvested via ultracentrifugation followed by washes to remove SDS as described above. Purified PG was digested with 50 µg/mL Cellosyl (gift from Hoechst, Frankfurt, Germany) in 20 mM sodium phosphate (pH 4.8) overnight at 37°C, followed by heating at 100°C for 10 min. Soluble muropeptides were harvested via brief centrifugation (20,000 *× g*, 1 min) and mixed with an equal volume of 0.5 M sodium borate (pH 9.0) and reduced with sodium borohydride (NaBH_4_) for 30 min at room temperature. The pH was adjusted to 3.5–4.5 with 20% phosphoric acid. The samples were stored at −20°C.

Separation of the reduced muropeptides by HPLC was described previously (57). The eluted muropeptides were monitored by measuring absorbance at 204 nm. Relative quantification of each muropeptide species was performed by integration of the peaks of the HPLC profile, and the muropeptides were grouped into classes according to structural similarities (57). Chromatograms are included in supplementary materials (Fig. S8).

## Data Availability

All data generated or analyzed during this study were included in this article and supplementary files.

## SUPPLEMENTAL MATERIAL

Supplemental material mbio.02184-25-s0001.docx Table S1 and S3 captions; Table S2; Fig. S1 to S8.

Table S1 mbio.02184-25-s0002.xlsx BS resistance rank of MG1655-S Δ*waaL* BP suppressor mutants and characterization of its derivative SBP mutants in ColE2 and P1 resistance.

Table S3 mbio.02184-25-s0003.xlsx Summary of muropeptide profile of MG1655-S Δ*waaL* grown in the absence or presence of BS.

ASM does not own the copyrights to Supplemental Material that may be linked to, or accessed through, an article. The authors have granted ASM a non-exclusive, world-wide license to publish the Supplemental Material files. Please contact the corresponding author directly for reuse.
