# Supplementary material for: Disruptions in outer membrane-peptidoglycan interactions enhance bile salt resistance in O-antigen-producing E. coli
Source: mBio. 2025 Aug 28;16(10):e02184-25. doi: 10.1128/mbio.02184-25 (PMC12505904; doi:10.1128/mbio.02184-25)
Supplement: Supplemental material — Table S1 and S3 captions; Table S2; Fig. S1 to S8. [file mbio.02184-25-s0001.docx]

**Supplementary Table 1. BS resistance rank of MG1655-S Δ*waaL* BP suppressor mutants and characterization of its derivative SBP mutants in ColE2 and P1 resistance.**

**Supplementary Table 2. Strains, plasmids, and oligonucleotides**

| **Bacterial strains** | | | | | | | |
| --- | --- | --- | --- | --- | --- | --- | --- |
| **Strains** | | | | | **Description** | **Source** | |
| MG1655 | | | | | Wild-type *E. coli* K-12 MG1655 | Lab stock | |
| MG1655-S | | | | | MG1655 with IS5I removed in *wbbL* | [1] | |
| MG1655 Δ*tolC* | | | | | MG1655 Δ*tolC::cat* | [1] | |
| MG1655 Δ*waaC* | | | | | MG1655 Δ*waaC::cat* | This work | |
| MG1655 Δ*waaP* | | | | | MG1655 Δ*waaP::cat* | This work | |
| MG1655 Δ*waaF* | | | | | MG1655 Δ*waaF::cat* | This work | |
| MG1655 Δ*waaB* | | | | | MG1655 Δ*waaB::cat* | This work | |
| MG1655 Δ*waaO* | | | | | MG1655 Δ*waaO::cat* | This work | |
| MG1655 Δ*waaG* | | | | | MG1655 Δ*waaG::cat* | This work | |
| MG1655 Δ*galU* | | | | | MG1655 Δ*galU::cat* | This work | |
| MG1655-S Δ*waaU* | | | | | MG1655-S Δ*waaU::neo* | [1] | |
| MG1655-S Δ*waaB* | | | | | MG1655-S Δ*waaB::neo* | This work | |
| MG1655-S Δ*waaF* | | | | | MG1655-S Δ*waaF::neo* | This work | |
| MG1655-S Δ*waaG* | | | | | MG1655-S Δ*waaG::neo* | This work | |
| MG1655-S Δ*waaO* | | | | | MG1655-S Δ*waaO::neo* | This work | |
| MG1655-S Δ*waaL* | | | | | MG1655-S Δ*waaL::frt* | [1] | |
| MG1655-S Δ*waaL* Δ*waaB* | | | | | MG1655-S Δ*waaL::frt* Δ*waaB::neo* | This work | |
| MG1655-S Δ*waaL* Δ*waaF* | | | | | MG1655-S Δ*waaL::frt* Δ*waaF::neo* | This work | |
| MG1655-S Δ*waaL* Δ*waaO* | | | | | MG1655-S Δ*waaL::frt* Δ*waaO::neo* | This work | |
| MG1655-S Δ*waaL* Δ*envZ* | | | | | MG1655-S Δ*waaL::frt* Δ*envZ::neo* | This work | |
| MG1655-S Δ*waaL* Δ*ompA* | | | | | MG1655-S Δ*waaL::frt* Δ*ompA::neo* | This work | |
| MG1655-S Δ*waaL* Δ*yhdP* | | | | | MG1655-S Δ*waaL::frt* Δ*yhdP::neo* | This work | |
| MG1655-S Δ*waaL* Δ*pstS* | | | | | MG1655-S Δ*waaL::frt* Δ*pstS::neo* | This work | |
| MG1655-S Δ*waaL* Δ*pqiB* | | | | | MG1655-S Δ*waaL::frt* Δ*pqiB::neo* | This work | |
| MG1655-S Δ*waaL* Δ*cydA* | | | | | MG1655-S Δ*waaL::frt* Δ*cydA::neo* | This work | |
| MG1655-S Δ*waaL* Δ*ompC* | | | | | MG1655-S Δ*waaL::frt* Δ*ompC::neo* | This work | |
| MG1655-S Δ*waaL* Δ*ompF* | | | | | MG1655-S Δ*waaL::frt* Δ*ompF::neo* | This work | |
| MG1655-S Δ*waaL* Δ*dsbA* | | | | | MG1655-S Δ*waaL::frt* Δ*dsbA::neo* | This work | |
| MG1655-S Δ*waaL* Δ*ugpB* | | | | | MG1655-S Δ*waaL::frt* Δ*ugpB::neo* | This work | |
| MG1655-S Δ*ugpB* | | | | | MG1655-S Δ*ugpB::neo* | This work | |
| MG1655-S Δ*waaL* Δ*pstS* Δ*ugpB* | | | | | MG1655-S Δ*waaL::frt* Δ*pstS::frt* Δ*ugpB::neo* | This work | |
| MG1655-S Δ*waaL* Δ*degP* | | | | | MG1655-S Δ*waaL::frt* Δ*degP::cat* | This work | |
| MG1655-S Δ*waaL* Δ*skp* | | | | | MG1655-S Δ*waaL::frt* Δ*skp::neo* | This work | |
| MG1655-S Δ*waaL* Δ*surA* | | | | | MG1655-S Δ*waaL::frt* Δ*surA::neo* | This work | |
| MG1655-S Δ*waaL* Δ*fkpA* | | | | | MG1655-S Δ*waaL::frt* Δ*fkpA::neo* | This work | |
| MG1655-S Δ*waaL* Δ*spy* | | | | | MG1655-S Δ*waaL::frt* Δ*spy::neo* | This work | |
| MG1655-S Δ*waaL-ompA*^Δ^*^CTD^* | | | | | MG1655-S Δ*waaL::frt -ompA*^Δ^*^CTD^::neo* | This work | |
| MG1655-S Δ*waaL* Δ*rcsF* | | | | | MG1655-S Δ*waaL::frt* Δ*rcsF::cat* | This work | |
| MG1655-S Δ*rcsF* | | | | | MG1655-S Δ*rcsF::cat* | This work | |
| MG1655-S Δ*waaL* Δ*lpp* | | | | | MG1655-S Δ*waaL::frt* Δ*lpp::neo* | This work | |
| MG1655-S Δ*waaL* Δ*ldtA* | | | | | MG1655-S Δ*waaL::frt* Δ*ldtA::neo* | This work | |
| MG1655-S Δ*waaL* Δ*ldtB* | | | | | MG1655-S Δ*waaL::frt* Δ*ldtB::neo* | This work | |
| MG1655-S Δ*waaL* Δ*ldtC* | | | | | MG1655-S Δ*waaL::frt* Δ*ldtC::neo* | This work | |
| MG1655-S Δ*waaL-lpp*^Δ^*^K58^* | | | | | MG1655-S Δ*waaL::frt-lpp*^Δ^*^K58^::neo* | This work | |
| SL1344 | | | | | *Salmonella enterica* SL1344 | Lab Stock | |
| SL1344 Δ*waaL* | | | | | SL1344 Δ*waaL::neo* | This work | |
| **Plasmids** | | | | |  |  | |
| **Plasmids** | | | | | **Description** | **Source** | |
| pSU2718 | Cloning plasmid, lac promoter, Chl^R^ | | | | | [2] | |
| pKD46 | Temperature sensitive plasmid expressing Red proteins, Amp^R^ | | | | | [2, 3] | |
| pCP20 | Plasmid expressing FLP flippase, Amp^R^ | | | | | [3] | |
| pKD4 | Plasmid carrying FRT flanked kanamycin resistant cassette, Amp^R^, Kan^R^ | | | | | [3] | |
| pKD3 | Plasmid carrying FRT flanked kanamycin resistant cassette, Amp^R^, Chl^R^ | | | | | [3] | |
| pWaaL | waaL CDS cloned from MG1655 into pSU2718 | | | | | [1, 3] | |
| pUgpB | *ugpB* CDS cloned from MG1655 into pSU2718 | | | | | This work | |
| pOmpA | *ompA* CDS cloned from MG1655 into pSU2718 | | | | | This work | |
| pOmpA^ΔCTD^ | *ompA* 1-189 and stop codon cloned from MG1655 into pSU2718 | | | | | This work | |
| pLpp | *Lpp* CDS cloned from MG1655 into pSU2718 | | | | | This work | |
|  |  | | | | |  | |
| **Oligos** | |  | | | |  | |
| **Description** | | | | **Sequence** | |  | |
| *waaC* KO F | | | GAACTCAACGCGCTATTGTTACAAGAGGAAGCCTGACGGgtgtaggctggagctgcttc | | | |  |
| *waaC* KO R | | | CAATGAATGAAGTTTAAAGGATGTTAGCATGTTTTACCTATGGGAATTAGCCATGGTCC | | | | |
| *waaF* KO F | | | TACATGGCCTGGCTGAATCGCGACGCATAAGAGCTCTGCgtgtaggctggagctgcttc | | | | |
| *waaF* KO R | | | GCCCATCGACGATGTTTTAACGATCAAAACCCGCATCCGATGGGAATTAGCCATGGTCC | | | | |
| *waaG* KO F | | | ACGCCAAAGTGTGGCAAGCGGCTCTTTTAATTCAACCATATGGGAATTAGCCATGGTCC | | | | |
| *waaG* KO R | | | GCCAGAAGATGCCCCTTCAGCTGACAGGAATGCACAATTgtgtaggctggagctgcttc | | | | |
| *waaO* KO F | | | AATGCTACCCTTATATCATTACTTTATAGTTTCCCAGTTATGGGAATTAGCCATGGTCC | | | | |
| *waaO* KO R | | | ACATAAGGTAATTATTTCTGCTATTTCCCGGAGGAAATAgtgtaggctggagctgcttc | | | | |
| *waaP* KO F | | | CATAATAAAGTTAGTTCCAGTACATACTAATAAATATTTATGGGAATTAGCCATGGTCC | | | | |
| *waaP* KO R | | | AAAGCCGCGGATATCATTACAGGTGGTTTAGATGGTTGAgtgtaggctggagctgcttc | | | | |
| *waaB* KO F | | | AATTAAGAAACTCAGTTTCCTGGAAAAACACCTGCTGCAATGGGAATTAGCCATGGTCC | | | | |
| *waaB* KO R | | | GTTAAAACAGTGAGTTTTACTCAATTAATGAGAGTTTCTgtgtaggctggagctgcttc | | | | |
| *galU* KO F | | | TGAACACGTTCAAAACACGAACAGTCCAGGAGAATTTAAGTGTAGGCTGGAGCTGCTTC | | | | |
| *galU* KO R | | | ATTGCTCAACGCCGTTTCGTGGATAACACCGATACGGATATGGGAATTAGCCATGGTCC | | | | |
| *envZ* KO F | | | tgcgcttctcgccacgaagttcatttgcccgtacgttattgctcatcgtcGTGTAGGCTGGAGCTGCTTC | | | | |
| *envZ* KO R | | | gtcgtgccctgcgcccgcgttaccggcactggcagccaggcgcgaatggaATGGGAATTAGCCATGGTCC | | | | |
| *ompA* KO F | | | ctcgttggagatattcatggcgtattttggatgataacgaggcgcaaaaaGTGTAGGCTGGAGCTGCTTC | | | | |
| *ompA* KO R | | | aaaggcaaaaaaaaccccgcagcagcggggtttttctaccagacgagaacATGGGAATTAGCCATGGTCC | | | | |
| *yhdP* KO F | | | tgccggggattttactgcttactggagccgcgctcgttgtgatcgctgccGTGTAGGCTGGAGCTGCTTC | | | | |
| *yhdP* KO R | | | tctttacgcggttggcgcaacacttcgttgatttgcggatcgtccagcggATGGGAATTAGCCATGGTCC | | | | |
| *pstS* KO F | | | tgcgtaccaccgtcgcaactgttgtcgccgcgaccttatcgatgagtgctGTGTAGGCTGGAGCTGCTTC | | | | |
| *pstS* KO R | | | ccgctactgtctttaatattggtcttccacgcagcgcgaacctgttcaacATGGGAATTAGCCATGGTCC | | | | |
| *pqiB* KO F | | | ataatggggaagccaaaatccagaaagtgaagaactggtctcccgtgtggGTGTAGGCTGGAGCTGCTTC | | | | |
| *pqiB* KO R | | | ttcggctctggatcttttttgtccttcgcttcaaataccagcgcgttactATGGGAATTAGCCATGGTCC | | | | |
| *cydA* KO F | | | tagtcgaactgtcgcgcttacagtttgccttgaccgcgatgtaccacttcGTGTAGGCTGGAGCTGCTTC | | | | |
| *cydA* KO R | | | gttttcaggctgcttgggccgaggcgtgcaaacttgaacattaagaacaaATGGGAATTAGCCATGGTCC | | | | |
| *ompC* KO F | | | aagtactgtccctcctggtcccagctctgctggtagcaggcgcagcaaacGTGTAGGCTGGAGCTGCTTC | | | | |
| *ompC* KO R | | | cccagagctacgatgttatcagtgttgatgccagcgtcacgagtgaactgATGGGAATTAGCCATGGTCC | | | | |
| *ompF* KO F | | | gcaatattctggcagtgatcgtccctgctctgttagtagcaggtactgcaGTGTAGGCTGGAGCTGCTTC | | | | |
| *ompF* KO R | | | cccacagcaacggtgtcgtctgaacctacgcccagtttgttgtcagaatcATGGGAATTAGCCATGGTCC | | | | |
| *dsbA* KO F | | | agaaccccctttgcaattaacacctatgtattaatcggagagagtagatcGTGTAGGCTGGAGCTGCTTC | | | | |
| *dsbA* KO R | | | aataaaaaaagcccgtgaatattcacgggctttatgtaatttacattgaaATGGGAATTAGCCATGGTCC | | | | |
| *ugpB* KO F | | | gcacaggcagtgacgaccattccgttctggcattctatggaaggggaactGTGTAGGCTGGAGCTGCTTC | | | | |
| *ugpB* KO R | | | ctgtggtgtcttcttaccggtccacacgctctccagctcttcatccacaaATGGGAATTAGCCATGGTCC | | | | |
| *degP* KO F | | | tctgaagaacacagcaattttgcgttatctgttaatcgagactgaaatacgtgtaggctggagctgcttc | | | | |
| *degP* KO R | | | cagattgtaaggagaaccccttcccgttttcaggaaggggttgagggagaatgggaattagccatggtcc | | | | |
| *skp* KO F | | | ttgcaatcgtcaacatgggcagcctgttccagcaggtagcgcagaaaaccGTGTAGGCTGGAGCTGCTTC | | | | |
| *pal* KO F | | | acaaagtgctgaaagggctgatgattgctctgcctgttatggcaattgcgGTGTAGGCTGGAGCTGCTTC | | | | |
| *pal* KO R | | | gcacgacggtttttggagtatgccgcttcgtcatgacccagtactgcaggATGGGAATTAGCCATGGTCC | | | | |
| *tolA* KO F | | | aaacgacaagctcaagcgggcgataattatttcagcagtgGTGTAGGCTGGAGCTGCTTC | | | | |
| *tolA* KO R | | | tgaacacttcatatactgcctggcttggtggtttcgggatATGGGAATTAGCCATGGTCC | | | | |
| *skp* KO R | | | ctgttgtaagcaacggcgtttgcatcaacaaccagatcgatatcctggctATGGGAATTAGCCATGGTCC | | | | |
| *fkpA* KO F | | | tgctggcgaccacaatggccgttgccctgcatgcaccaatcacttttgctGTGTAGGCTGGAGCTGCTTC | | | | |
| *fkpA* KO R | | | gcatcagccttcggcgctggtttcacatccagcagctctacgtcaaacacATGGGAATTAGCCATGGTCC | | | | |
| *surA* KO F | | | TGATTTACCACGTAATCCGCAGTGCGGTTAATTGAAATGGAAAAAGTATGGTGTAGGCTGGAGCTGCTTC | | | | |
| *surA* KO R | | | AGTGATCACAACACGTTGGGTTTTAACCATTAGTTGCTCAGGATTTTAACATGGGAATTAGCCATGGTCC | | | | |
| *spy* KO F | | | gtttgttgcctctaccctggctcttggcgcggctaacctgGTGTAGGCTGGAGCTGCTTC | | | | |
| *spy* KO R | | | ttgccgctggacgttctgtcagacgcttctcaaaattagcATGGGAATTAGCCATGGTCC | | | | |
| *rcsF* KO F | | | TTCGCCGTGATGTTAAGCGCAGAACCGATACATACAGCCTGACGATAGCAGTGTAGGCTGGAGCTGCTTC | | | | |
| *rcsF* KO R | | | ATGCGTGCTTTACCGATCTGTTTAGTAGCACTCATGCTAAGCGGCTGTTCATGGGAATTAGCCATGGTCC | | | | |
| *Lpp* KO F | | | atgaaagctactaaactggtactgggcgcggtaatcctgggttctactctGTGTAGGCTGGAGCTGCTTC | | | | |
| *Lpp* KO R | | | ttacttgcggtatttagtagccatgttgtccagacgctggttagcacgagATGGGAATTAGCCATGGTCC | | | | |
| *ldtA* KO F | | | ttgctcatttgctctgctttttgccagccatactagcctgGTGTAGGCTGGAGCTGCTTC | | | | |
| *ldtA* KO R | | | taatttgcacaggcattcccgatcgacgttgcaacgcagcATGGGAATTAGCCATGGTCC | | | | |
| *ldtB* KO F | | | tcgcagcggccttcgctgttgtcggcttttgcagtaccgcctctgcggtaGTGTAGGCTGGAGCTGCTTC | | | | |
| *ldtB* KO R | | | ttaatcgcttcatcaagaacaacctggtcaacatctggctgaccggtcacATGGGAATTAGCCATGGTCC | | | | |
| *ldtC* KO F | | | ttctcgctggctaacgttttttacgttcgccgctgccgtgGTGTAGGCTGGAGCTGCTTC | | | | |
| *ldtC* KO R | | | gacggcggacatccaccggcatcccggaacggacatccatATGGGAATTAGCCATGGTCC | | | | |
| *ompA* CTD+his6 KO F | | | tcgtccggacaacggcatgctgagcctgggtgtttcctacCATCATCACCATCACCACTAAGTGTAGGCTGGAGCTGCTTC | | | | |
| *Lpp K58* KO F | | | cagctcgtgctaaccagcgtctggacaacatggctactaaataccgctaaGTGTAGGCTGGAGCTGCTTC | | | | |
| *Lpp K58* KO R | | | acaaaaaaaatggcgcacaatgtgcgccatttttcacttcacaggtactaATGGGAATTAGCCATGGTCC | | | | |
| *Lpp* cloning F | | | AATTGGTACCatgaaagctactaaactggtactg | | | | |
| *Lpp* cloning R | | | aattTCTAGAttacttgcggtatttagtagccatg | | | | |
| *ugpB* cloning F | | | aattGGTACCatgaaaccgttacattatacagcttc | | | | |
| *ugpB* cloning R | | | aattTCTAGAttaagacttcgtcgatttctcaaag | | | | |
| *ompA* cloning F | | | aattGGTACCatgaaaaagacagctatcgcg | | | | |
| *ompA* cloning R | | | aattTCTAGAttaagcctgcggctgagttac | | | | |
| *ompA1-189-his6* cloning F | | | aattGGTACCatgaaaaagacagctatcgcg | | | | |
| *ompA1-189-his6* cloning R | | | aattTCTAGAttaGTGGTGATGGTGATGATGgtaggaaacacccaggctc | | | | |
| *SL1344 waaL KO F* | | | catcattaacgttaaataaagagaaatggaagccgatctggaataaagcgGTGTAGGCTGGAGCTGCTTC | | | | |
| *SL1344 waaL KO R* | | | aaaccggtaatgataccaatttgagcaatatcgacctgttcaaaattgccATGGGAATTAGCCATGGTCC | | | | |

**Supplementary Table 3. Summary of muropeptide profile of MG1655-S Δ*waaL* grown in the absence or presence of BS.**


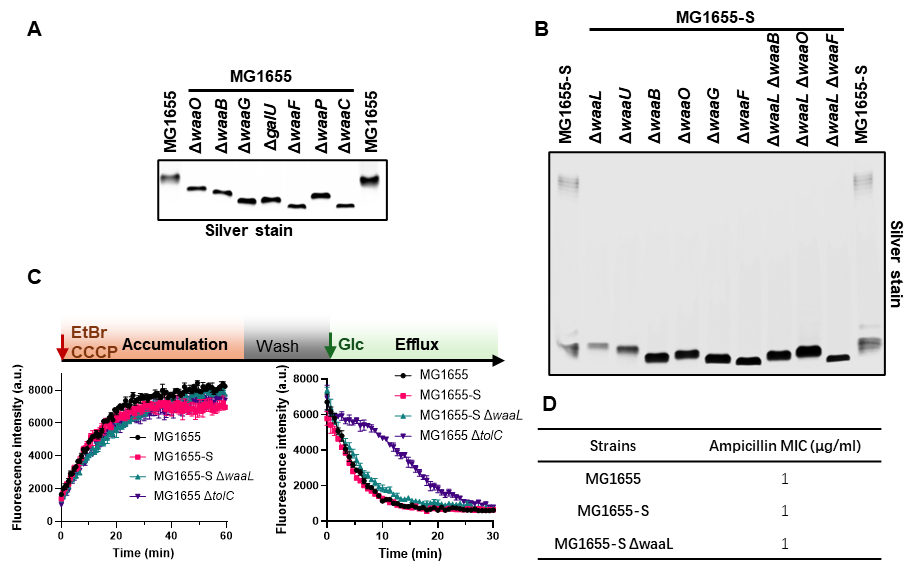


**S1 Fig. Validation of LPS core truncations mutants of MG1655 derivative strains and effect of periplasmic UndPP-OAg accumulation on multidrug efflux activity. (A&B)** LPS silver staining of proteinase K-treated whole cell lysates of indicated MG1655 derivative strains. (**C**) Cellular EtBr accumulation and efflux assay of indicated MG1655 derivative strains. See Methods and Materials. (**D**) MIC of ampicillin determined for indicated *E. coli* K-12 strains.


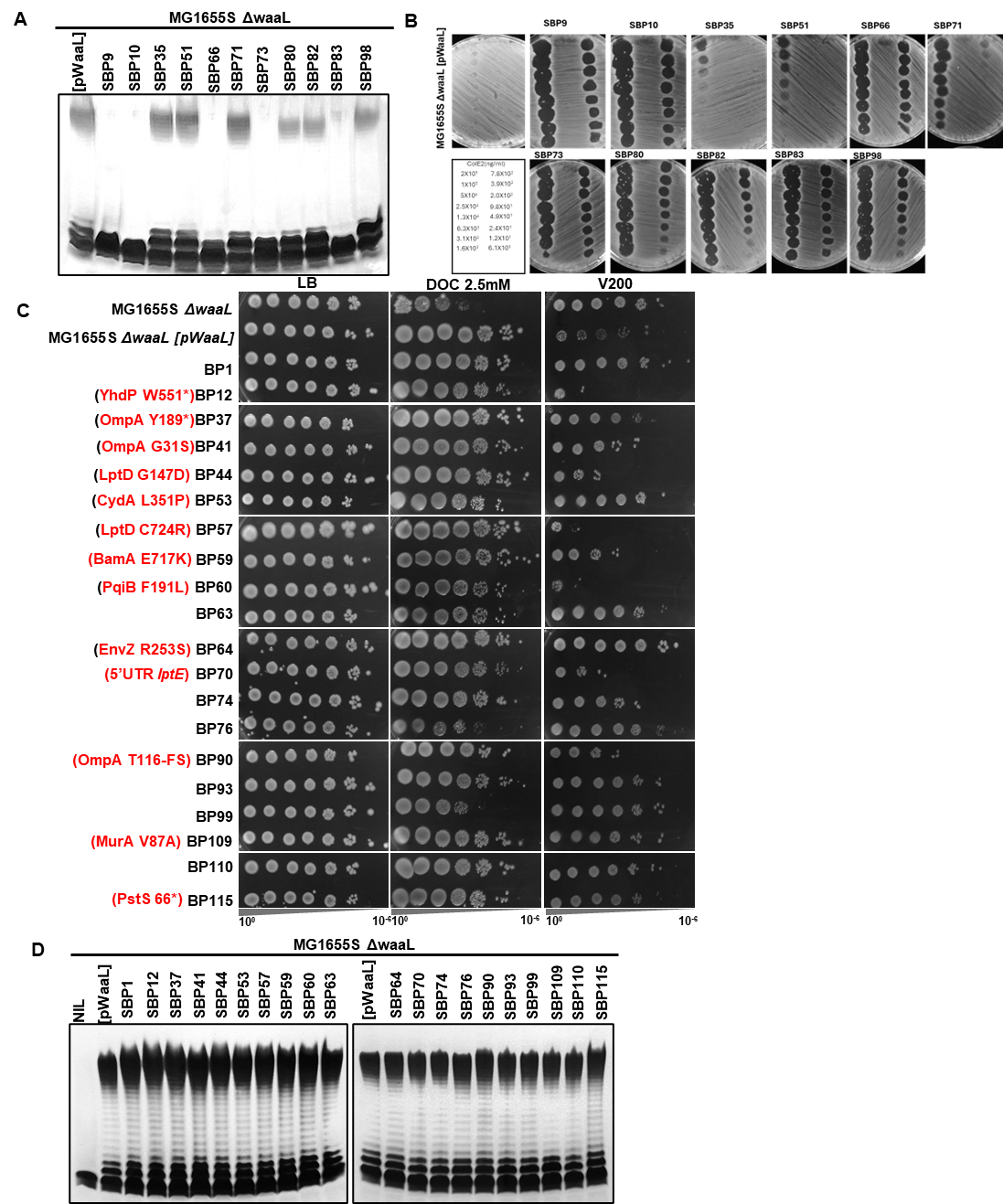


**S2 Fig. Characterisation of LPS profile of SBP mutants and their corresponding BP suppressor mutants’ BS resistance.** (**A**) LPS silver staining of proteinase K-treated whole cell lysates of indicated MG1655-S Δ*waaL* SBP mutant strains. (**B**) Colicin E2 sensitivity assay of indicated MG1655-S Δ*waaL* SBP mutant strains. Colicin E2 (5 μl, at indicated concentrations) was spotted onto LBA plate pre-spread with cultures of indicated MG1655-S Δ*waaL* SBP mutant strains. Sensitivity to Colicin E2 was shown as inhibited bacterial growth. (**C**) BS and vancomycin (200 μg/ml) sensitivity assay to indicated MG1655-S Δ*waaL* BP mutant strains. Mutations mapped through whole genome sequencing was indicated in red. (**D**) LPS silver staining of proteinase K-treated whole cell lysates of indicated MG1655-S Δ*waaL* SBP mutant strains.


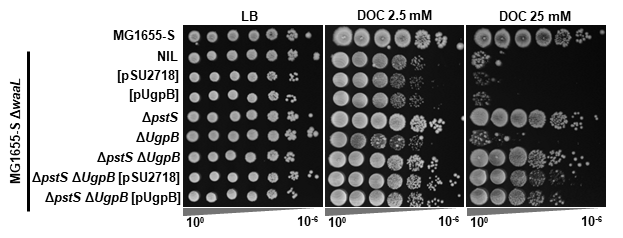


**S3 Fig. Investigation the role of UgpB in conferring BS resistance to MG1655-S Δ*waaL.*** Indicated MG1655-S derivative strains harbouring indicated expression construct were assayed for BS resistance with 2.5 mM and 25 mM DOC.


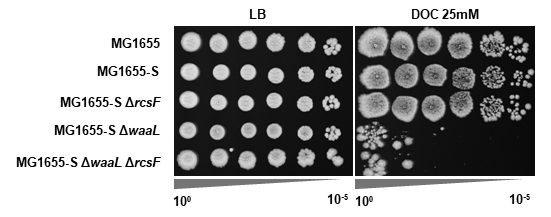


**S4 Fig. Investigation the role of RcsF in conferring BS resistance to MG1655-S Δ*waaL.*** Indicated MG1655-S derivative strains were assayed for BS resistance with 25 mM DOC.


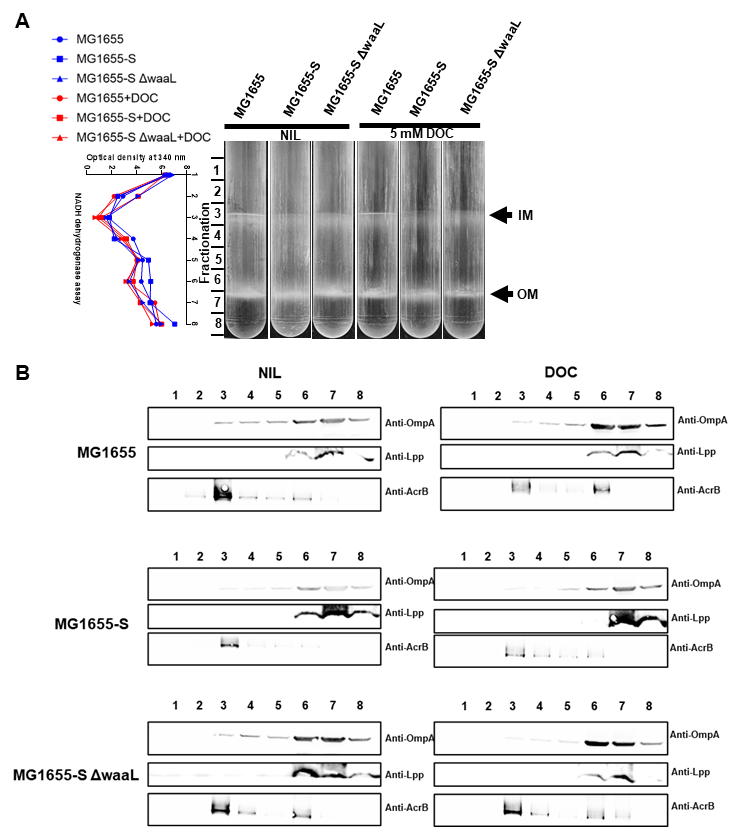


**S5 Fig. Membrane localisation of Lpp in MG1655-S Δ*waaL* grown in the presence of BS.** Bacterial cell membranes were isolated from indicated bacterial cell cultures with or without 5 mM DOC (detailed in Materials and Methods). IM and OM (marked in **A**) were separated via sucrose gradient density centrifugation (**A**). The separated membrane gradient was fractionated (1 ml) indicated on the left side. Samples of fractions were taken to evaluate the NADPH dehydrogenase activity to validate the successful separation of IM from OM, with lower OD_340_ reading correlates to higher activity of NADPH dehydrogenase detected in the fraction (**A left curve**). Validated fractions were then subjected to Western immunoblotting (**B**) to investigate the membrane localisation of Lpp via anti-Lpp antibodies. Anti-OmpA and anti-AcrB were used as a reference for OM and IM localisations, respectively.


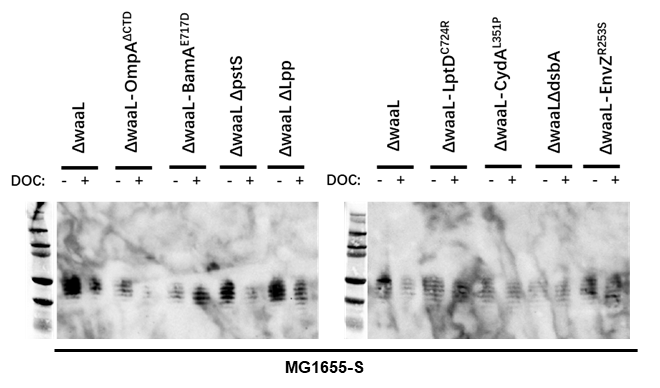


**S6 Fig. Confirmation of presence of UndPP-OAg in the MG1655-S** Δ***waaL* derivative strains via Western immunoblotting.** MG1655-S Δ*waaL* SBP mutant strains were allowed to grow to OD_600_ of 0.8, and DOC (25 mM) was added for another 30 min growth. MG1655-S Δ*waaL* showed clear lysis, and all suppressor strains showed mild lysis. Proteinase K-treated bacterial cell lysates were prepared and then subjected to Western immunoblotting with anti-O16 antibodies. UndPP-OAg was detected as ladder pattern bands.


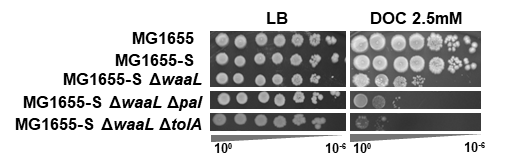


**S7 Fig. Investigation the role of Tol-Pal in conferring BS resistance to MG1655-S Δ*waaL.*** Indicated MG1655-S derivative strains were assayed for BS resistance with 2.5 mM DOC.

**S8 Fig. Muropeptide profile of MG1655-S Δ*waaL* grown in the presence or absence of BS**. Chromatography of muropeptide derived from MG1655-S Δ*waaL* grown in the absence (**A**) or presence (**B**) of DOC were isolated and processed according to Materials and Methods, the processed muropeptides were profiled by HPLC as detailed in Materials and Methods. Major peaks corresponding to Tetra, Tetra-Tetra and TriLysArg (Lpp–PG linkage) are indicated according to Glauner (4).

1. Qin J, Hong Y, Morona R, Totsika M. O antigen biogenesis sensitises Escherichia coli K-12 to bile salts, providing a plausible explanation for its evolutionary loss. PLoS Genet. 2023;19(10):e1010996. Epub 2023/10/04. doi: 10.1371/journal.pgen.1010996. PubMed PMID: 37792901; PubMed Central PMCID: PMCPMC10578602.

2. Martinez E, Bartolome B, de la Cruz F. pACYC184-derived cloning vectors containing the multiple cloning site and lacZ alpha reporter gene of pUC8/9 and pUC18/19 plasmids. Gene. 1988;68(1):159-62. Epub 1988/08/15. doi: 10.1016/0378-1119(88)90608-7. PubMed PMID: 2851489.

3. Datsenko KA, Wanner BL. One-step inactivation of chromosomal genes in Escherichia coli K-12 using PCR products. Proceedings of the National Academy of Sciences of the United States of America. 2000;97(12):6640-5. doi: DOI 10.1073/pnas.120163297. PubMed PMID: WOS:000087526300074.

4. Glauner B. Separation and quantification of muropeptides with high-performance liquid chromatography. Anal Biochem. 1988;172(2):451-64. Epub 1988/08/01. doi: 10.1016/0003-2697(88)90468-x. PubMed PMID: 3056100.
